# An Augmented Multiple Imputation Particle Filter for River State Estimation With Missing Observation

**DOI:** 10.3389/frobt.2021.788125

**Published:** 2022-02-18

**Authors:** Z. H. Ismail, N. A. Jalaludin

**Affiliations:** ^1^ Centre for Artificial Intelligence and Robotics, Universiti Teknologi Malaysia, Kuala Lumpur, Malaysia; ^2^ Faculty of Engineering Technology, Universiti Tun Hussein Onn Malaysia, Batu Pahat, Malaysia

**Keywords:** data assimilation, particle filter, smooth variable structure filter, marine observation, state estimation

## Abstract

In this article, a new form of data assimilation (DA) method namely multiple imputation particle filter with smooth variable structure filter (MIPF–SVSF) is proposed for river state estimation. This method is introduced to perform estimation during missing observation by presenting new sets of data. The contribution of this work is to overcome the missing observation, and at the same time improve the estimation performance. The convergence analysis of the MIPF–SVF is discussed and shows that the method depends on the number of particles and imputations. However, the number of particles and imputations is influenced by the error difference in the likelihood function. By bounding the error, the ability of the method can be improved and the number of particles and computational time are reduced. The comparison between the proposed method with EKF during complete data and multiple imputation particle filter shows the effectiveness of the MIPF–SVSF. The percentage improvement of the proposed method compared to MIPF in terms of root mean square error is between 12 and 13.5%, standard deviation is between 14 and 15%, mean absolute error is between 2 and 7%, and the computational error is reduced between 73 and 90% of the length of time required to perform the estimation process.

## Introduction

The prediction of the river state is important in the hydrology, water resource management, and ecosystem rehabilitation. The knowledge of river flow characteristics is useful for flood forecasting, reservoir operations, and watershed modeling (Sichangi et al., 2018). In flood forecasting, the predicted state is use to produce alerts of the incoming flood to prevent damages to human life, properties and environment (Jain et al., 2018). Besides that, the information from the state estimation is used in controlling the outflows of reservoir during low flows day of river and also rapid flows resulting from dam-break that may cause catastrophy to the environment and massive losses to life and property (Adnan et al., 2017), (Cao et al., 2019). The water flow can also be used in watershed modeling that is used to manage the water by channeling the water from any sources into a single larger body of water such as a larger river (Aswathy et al., 2016). The management and planning of the water source is important for the increasing of the water demand in the next few years due to the increasing population growth, urbanization, industrial use, irrigation needs, and water-intensive agriculture (Tinka et al., 2013). This includes the planning of the water projects, irrigation systems, hydropower system, and optimized utilization of water resources (Adnan et al., 2017). The information of the river discharge (flow) is necessary in the management procedure and the variation of the hydrologic cycle is related to the climate change, land use, and water use (Bjerklie et al., 2018). The river discharge is one of the climate variables by the Global Climate Observing System (Tarpanelli et al., 2019). The prediction of the variables that represent the water flow regime helps in the ecosystem rehabilitation program that seeks to safeguard or restore indigenous ecosystems by manipulating the river flow (Blythe and Schmidt, 2018). The estimation of the river state can be carried out using the data assimilation (DA) method. The DA method is a mathematical technique that combines observation data with the system model and creates the updated model state while maintaining the parameter of the model (Li et al., 2014). The updated model state is defined as the probability density function (pdf) that is based on Bayes’ theorem and known as the new posterior pdf (Smith et al., 2013). The new state is acquired whenever there are new observations and uses them to initiate the next model forecast as reported in a study (He et al., 2014). The DA method is desired to optimally and consistently estimated, even if the noisy readings arrive sequentially in time (Liu and Gupta, 2007). The DA technique is accessible in two classes namely, variational and sequential (Gadsden et al., 2014).

The variational method is based on the optimum theory of control. Optimization is carried out on the associated parameters by minimizing the cost function that influences the model to misfit the information. The examples of this technique are variational data assimilation (VAR), evolutionary data assimilation (EDA), and maximum likelihood ensemble filter (MLEF) (Abaza et al., 2014). The VAR method makes estimates by minimizing the cost function which measures the difference between the model estimate, the observation, and the associated uncertainties. The gradient-based optimization algorithm is used to adjust the model states and parameters of the model and receive the appropriate estimate for the measurement (Kim et al., 2014). Besides that, the EDA utilizes a multi-objective evolutionary strategy to continually develop the set of model states and parameters where the model error and penalty function minimization for each assimilation time step is determined adaptively. This is done to enhance the convergence of parameters and lead to excellent estimation outcomes (Bertino et al., 2003; Solonen et al., 2014). Another type of variational method is the MLEF, which combines the VAR and the ensemble Kalman filter (EnKF). This method maintains the strength of both method and capable to handle nonlinear model dynamics as well as nonlinear observation. However, in some cases the performance of the MLEF may deteriorate due to consistency of the observation (Rafieeinasab et al., 2014). Besides that, the sequential methods use a probabilistic framework and estimate the whole system state sequentially by propagating information only forward in time. This method does not require an adjoint model and makes it easy to adapt with the model (Rigatos, 2012). The sequential-based technique is frequently used in estimation compared to the variational method, since the prior state is updated with the new observation available and the process is performed sequentially (Arulampalam et al., 2002). The examples of this method are extended Kalman filter in (Li et al., 2014), ensemble Kalman filter (EnKF) in (Rafieeinasab et al., 2014), unscented Kalman filter (UKF) (Pintelon et al., 2017), cubature Kalman filter (Liu and Gupta, 2007), and particle filter (PF) (Ugryumova et al., 2015). The EKF is commonly used sequentially DA method due to easy implementation, but depends strongly on the accuracy of the system linearization that is performed using Taylor series expansion (Li et al., 2014). This technique shows excellent efficiency in low nonlinearity and diverges in higher nonlinear cases (Zhang et al., 2015). For a highly nonlinear system, the estimation can be carried out using the EnKF that offers estimation without linearization (Rafieeinasab et al., 2014). This method involves a large sample size to represent the number of samples or ensemble members for precise estimation that can be produced with Monte Carlo method, Latin hypercube sampling, and moment equation (Kang, 2013; Gadsden et al., 2014; Wang et al., 2017). The samples’ mean and covariance are used to perform updating in the estimation process (Rafieeinasab et al., 2014). However, the large sample size may cause for computational demand (Gadsden et al., 2014). Another form of EnKF is the ensemble square root filter (EnSRF) that does not require the observation to be perturbed as the standard implementation of the EnKF. The algorithm of this method has been demonstrated to be as fast as the EnKF and more precise for a specified ensemble size than EnKF (Liu et al., 2017).

The UKF is more appropriate for application for low computational and high accuracy. This method includes the sigma points derived from the unscented transformations (Ding et al., 2015). The sigma points are propagated through the system model and the related weight factor during the estimation process and produce new sets of sigma points, which are subsequently used in the calculation of the projected states (Pintelon et al., 2017). However, this method relies on the precise of prior noise distribution. Wrong prior value can lead to large estimation errors or divergence of errors (Mao et al., 2017). Another type of sequential DA method is the PF, which does not involve system linearization and includes a number of particles during prediction (Hu et al., 2012). Each particle represents the estimated state with its associated likelihood that is determined using the residual between the simulated output and observation (Solonen et al., 2014). Large numbers of particles provide the chance of good estimation, but the computational time will increase. Another factor to consider when using PF is the issue of degeneration and dimensionality curse that can influence the estimation (Ugryumova et al., 2015). All the abovementioned DA methods were implemented in the river state estimation study. The modification of the methods may improve the performance of the method and the result obtained. In a study (Cao et al., 2019), the particle filter is modified by considering the system condition and the particle weighting procedure. The modification has improved the estimation result. Besides that, the modification can also be made by combining two or more methods such as the combination of the PF with smooth variable structure filter (SVSF). The SVSF produce estimated state and state error covariance, which is used to formulate the proposal distribution for the PF to generate particles (Ogundijo et al., 2016). The SVSF is robust to modeling errors and uncertainties (Feng et al., 2011). This method produces better estimated result than the PF only. Besides that, the SVSF also combined with the cubature Kalman filter (CKF). The CKF offers the nearest known approximation to the Bayesian filter in the sense of maintaining second-order information contained in the Gaussian assumption of noisy measurements. This method also does not need Jacobians and therefore applies to a wide range of issues. The accuracy of CKF and the stability of the SVSF ensure good estimation results (Liu and Gupta, 2007).

In this work, the estimated river system flow and stage, and also the velocity of the sensor are inspired from (Tinka et al., 2013) and (Zhang et al., 2014). These values can be obtained using the previously mentioned DA method. However, the missing observation data can be problematic that will affect the estimation process (Ismail and Jalaludin, 2016). The missing data can be handled using modern method or traditional method. The modern method is represented by the maximum likelihood and multiple imputations, while the traditional techniques are deletion and mean imputation techniques (Gadsden et al., 2012). It is noted that the multiple imputation particle filter (MIPF) is introduced to deal with this problem by randomly drawn values known as imputations to replace the missing information and then uses the particle filter to predict nonlinear state as reported in (Habibi, 2007). However, the addition of the new data burdens the estimation process. Therefore, the SVSF is introduced to bound the error difference and assist in the estimation process.

In this article, the convergence analysis of the MIPF–SVSF in estimating the river flow and stage from the initial condition is presented. The article is constructed as follows: the system model, observation model, and the state space model for estimation process are presented in *Modelling the Marine River Flow*. *Problem Formulation* briefly explains the effect of the missing observation during estimation. Then, the proposed algorithm for estimation with missing data is described in the *Proposed Data Assimilation Approach*. The convergence analysis is presented in *Convergence Analysis* that includes almost sure convergence and convergence of the mean square error. In *Results and Discussion*, the details on estimation process and numerical simulations are discussed and finally conclusions are presented in *Conclusion*.

## Modeling the Marine River Flow

The river flow model can be represented by one or two-dimensional Saint–Venant equations (Tinka et al., 2013) depending on the characteristics of the water flow. If the flow is in one-dimensional, the 1-D Saint–Venant equations is considered. However, if the flow is not one-dimensional, which may happen in flood plains or in large rivers, the 2-D Saint–Venants equation is more suitable to be applied (Litrico and Fromion, 2009). Besides that, the representation of the observation is referring to the movement of the sensor since the Lagrangian sensor is used in this research (Tinka et al., 2013). The combination of the system model and the observation is represented by the state-space model and used in the DA method.

### System Model and Observation Model

By considering one-dimensional flow of the river without any uncontrolled release of water flow, the system model is represented by 1-D Saint–Venant equations. These two equations coupled are first order hyperbolic partial differential equations (pde) derived from the conservation of mass and momentum. By considering a prismatic channel that have same cross-section throughout the length of channel with no lateral inflow, the equation is represented as (Tinka et al., 2013).

$$T\frac{\partial H}{\partial t}+\frac{\partial Q}{\partial x}=0$$ (1)

$$\frac{\partial Q}{\partial t}+\frac{\partial }{\partial x}\left(\frac{Q^{2}}{A}\right)+\frac{\partial }{\partial x}\left(gh_{c}A\right)=gA\left(S_{o}-S_{f}\right)$$ (2)

$$S_{f}=\frac{m^{2}Q^{2}P^{\frac{4}{3}}}{A^{\frac{4}{3}}}$$ (3)

where *A* is the cross section, *Q* is the discharge or flow, L is the river reach, *T* is the free surface width, *D* is the hydraulic depth, *S*_*f*_ is the friction slope, *S*_*o*_ is the bed slope, *F*_*g*_ is the gravitational acceleration, *h*_*c*_ is the distance of the centroid of the cross section from the free surface, *P* is the wetted perimeter, and *m* is the Manning roughness coefficient.

### Observation Model

The system observation is represented by the velocity of the flow as measured by the sensors. The relation between the velocity of the sensor and the flow at the corresponding cross-section relies on assumptions made about the profile of the water velocity that considered as the observation model. The profile is the combination of the average velocity in the transverse and vertical direction. In transverse direction, the surface velocity profile is assumed to be quartic, and the von Karman logarithmic profile is assumed in the vertical direction. By considering a particle moving at a distance *y* from the center line and *z* from the surface, the relation between the particle’s velocity and the water flow is represented by the following equations (Tinka et al., 2013)

$$v_{p}\left(y,z\right)=F_{T}\left(y\right)F_{v}\left(z\right)\frac{Q}{A}$$ (4)

with

$$F_{T}\left(y\right)=A_{q}+B_{q}{\left(\frac{2y}{w}\right)}^{2}+C_{q}{\left(\frac{2y}{w}\right)}^{4}$$ (5)

$$A_{q}+B_{q}+C_{q}=0$$ (6)

$$A_{q}+\frac{B_{q}}{3}+\frac{C_{q}}{5}=1$$ (7)

$$F_{v}\left(z\right)=1+\left(\frac{0.1}{K_{v}}\right)\left(1+\log\left(\frac{z}{d}\right)\right)$$ (8)

where *w* is the channel width; *d* is the water depth; *A*_*q*_, *B*_*q*_, and *C*_*q*_ are constants; and *K*_*v*_ is the Von Karman log constant.

### State Space Representation

During estimation process, the system and observation equation is represented as a state-space model that comprises of the parameters of the model, observation, system noise, and measurement noise. The channel is discretized into *n* cells with each cell and has same length. In the nonlinear system state estimation, the initial conditions and the boundary conditions of the system are required as the inputs. The uncertainties of the model and also the inaccuracies of the inputs measurements are considered as the system noise $v_{t}$ . While the measurement noise, $\varepsilon_{t}$ represent the errors and uncertainties of the measurements. Both noises are represented by the zero mean Gaussian error. The state-space model for the estimation is described as follows

$$X_{t+1}=f\left(X_{t},u_{t},\upsilon_{t}\right)$$ (9)

$$W_{t}=g\left(X_{t},\varepsilon_{t},t\right)$$ (10)

where *X*_*t*_ is the state vector at time *t*

$$X_{t}={\left(Q_{2}^{t},\ldots ,Q_{n_{c}}^{t},H_{1}^{t},\ldots ,H_{n_{c}-1}^{t}\right)}^{T}$$ (11)

and the input *u*_*t*_ contains the boundary conditions, i.e., the upstream flow and downstream stage.

$$u_{t}={\left(Q_{1}^{t},H_{n_{c}}^{t}\right)}^{T}$$ (12)

where $Q_{i}^{t}$ and $H_{i}^{t}$ are the flow and stage at cell *i* at time *t*, respectively, and *n*_*c*_ is number of cells used for the discretization of the channel. Since the system is observed by *n*_*k*_ sensors, Eq. 10 can be reformulated into

$$W_{t}=\left(\begin{array}{c} g_{1}\left(X_{t},\varepsilon_{t,1},t\right)\\ \vdots \\ g_{n_{k}}\left(X_{t},\varepsilon_{t,n_{k}},t\right) \end{array}\right)=\left(\begin{array}{c} W_{t,1}\\ \vdots \\ W_{t,n_{k}} \end{array}\right)\text{for}k=1\ldots n_{k}$$ (13)

where $W_{t}$ denotes the noisy observation of the state *X*_*t*_ such that the $\varepsilon_{t,k}$ is an independent and identically distributed (i.i.d.) measurement noise and *g*_*k*_ is the measurement transformation for sensor *k*.

## Problem Formulation

The Bayesian theorem used by the DA method is represented as follows (Crisan and Doucet, 2002).

$$p\left(X_{t}|W_{1:t}\right)=\frac{p\left(W_{t}\text{|}X_{t}\right)p\left(X_{t}\text{|}W_{1:t-1}\right)}{p\left(W_{t}\text{|}W_{1:t-1}\right)}$$ (14)

where $X_{t}$ is the system state at time *t*, $W_{t}$ is the observation at time *t*, $p\left(X_{t}|W_{1:t}\right)$ is the posterior probability of state, X at time *t* given observation, W from time one to time *t*, $p\left(W_{t}\text{|}X_{t}\right)$ is the likelihood function of state X at time *t* given observation W at time *t*, $p\left(X_{t}\text{|}W_{1:t-1}\right)$ is the prior probability of state X at time *t* given observation W from time one to time *t*-1, $p\left(W_{t}\text{|}W_{1:t-1}\right)$ is the normalizing constant.

In this theorem, the observation data $W_{t}$ is used to adjust the likelihood function. The adjusted likelihood function is used to modify the prior probability to obtain the desired posterior probability that represents the estimated state. The normalizing constant in this theorem is represented as (Ugryumova et al., 2015).

$$p\left(W_{t}|W_{1:t-1}\right)=\int p\left(W_{t}|X_{t}\right)p\left(X_{t}|W_{1:t-1}\right)dX_{t}$$ (15)

where all parameters are defined in Eq. 14. Based on Eqs 14, 15, the posterior probability is very much depending on the likelihood function $p\left(W_{t}\text{|}X_{t}\right)$ . This function is represented as follow (Mukherjee and Sengupta, 2010)

$$p\left(W_{t}\text{|}X_{t}\right)=p\left(W_{t}-g\left(X_{t}\right)\right)$$ (16)

where $W_{t}$ and $X_{t}$ are defined in Eq. 14, and $g\left(X_{t}\right)$ represent the estimation of the observation using the predicted states. The difference between the observation and the estimated observation is considered in this function. This error affects the likelihood function, and thus influences the prediction result.

In this research, several types of observation namely *y* and *z* positions of the sensors, and the velocity of the sensors are considered. The positions of the sensors are used in determining the estimated observation using Eqs 4–8. Next, the obtained estimated velocity is compared with the measured velocity of the sensors and form the likelihood function for this case. Since the likelihood function is important in estimation, the continuous observations from the sensors are desired to secure this function throughout the estimation process. In the event of missing observation data, the likelihood function is affected, and thus limits the ability of the standard DA method. Therefore, the MIPF method is introduced to perform estimation with new input data that replace the missing data. The availability of the observations is checked at each time instance. The missing data are handled by introducing a random indicator variable, *R*_*t,k*_ (Abaza et al., 2014).

$$R_{t,k}\left\{ \begin{array}{l} 0:\;\;\text{Observation}\;\text{is}\;\text{missing}\;\text{from}\;\text{sensor}\;k\;\text{at}\;\text{time}\;t\\ 1:\;\;\text{Observation}\;\text{is}\;\text{available}\;\text{from}\;\text{sensor}\;k\;\text{at}\;\text{time}\;t \end{array}\right.$$

Consider the overall observation $W_{t,k}$ comprises both available and missing data from all sensors. The observation at time instance *t* for all sensors *k* = 1…,*n*_*k*_ with $R_{t,k}=0$ is defined as the missing information set $\Xi_{t}$ , while the available information set ${\text{Ψ}}_{t}$ is the observation for all sensors *k* = 1,…, *n*_*k*_ such that $R_{t,k}=1$ .

The introduction of the new data may affect the error difference between the observation from the new data and the estimated observation. Therefore, the SVSF method that is robust and stable in the estimation process is introduced to handle this problem. The combination of the MIPF and SVSF capable of handling state estimation with missing information and error differences problem.

During missing information, several random observations or imputations is introduced in the estimation process. The imputations are drawn from the proposal function (Chai and Draxler, 2014)

$$\Xi_{t}^{j}∼\varphi \left(\Xi_{t}\text{|}\Psi_{0:t}\right)=\sum_{i=1}^{N}{\tilde{\omega }}_{t}^{i}p\left(\Xi_{t}|{\tilde{X}}_{t}^{i}\right)\;\;\text{for}\;i=1,\ldots ,N\;\text{and}\;j=1,\ldots ,M$$ (17)

where $\Xi_{t}$ represent all missing observations at time *t*, ${\text{Ψ}}_{0:t}$ represent all available observation from time 0 to time *t*, ${\left\{{\tilde{\omega }}_{t}^{i},{\tilde{X}}_{t}^{i}\right\}}_{i\;=1}^{N}$ is the particle set with no regard of missing data, *N* is the total number of particles and *M* is the total number of imputations. Next, the imputations are reformulated into the imputed data sets of

$$U_{t}^{j}=\left\{\Xi_{t}^{j},\Psi_{t}\right\}$$ (18)

where $\Xi_{t}^{j}$ represent all missing observation during *j*th imputation and time *t*, and $\Psi_{t}$ represents all available observation at time *t*. The imputed sets are used in determining the posterior probability density that represent as

$$p\left(X_{t}|\Psi_{0:t}\right)=\int p\left(X_{t}|W_{0:t-1},\Psi_{t}\right)p\left(\Xi_{t}|\Psi_{0:t}\right)d\Xi_{t}$$ (19)

where $X_{t}$ is the system state at time *t*, $W_{0:t-1}$ is the complete observation, $\Xi_{t}$ and ${\text{Ψ}}_{0:t}$ are defined in Eq. 17, and ${\text{Ψ}}_{t}$ is defined in Eq. 18. Considering the Monte Carlo approximation, the probability density can be written as

$$p\left(X_{t}|\Psi_{0:t}\right)\approx \frac{1}{M}\sum_{j=1}^{M}p\left(X_{t}|W_{0:t-1},U_{t}^{j}\right)$$ (20)

where M is defined in Eq. 17, $U_{t}^{j}$ is defined in Eq. 18, and $X_{t}$ , ${\text{Ψ}}_{0:t}$ , and $W_{0:t-1}$ , are defined in Eq. 19. For each data set $U_{t}^{j}$ , the probability density from particle filtering is written as follows:

$$p\left(X_{t}|W_{0:t-1},U_{t}^{j}\right)\approx \sum_{i=1}^{N}\omega_{t}^{j,i}\delta \left(X_{t}-X_{t}^{j,i}\right)$$ (21)

where $X_{t}^{j,i}$ is the system state at *i*th particle and *j*th imputation at time instance t, $\omega_{t}^{j,i}$ is the related weight. By substituting Eq. 21 into Eq. 20, the overall representation of the desired posterior probability density is represented as

$$p\left(X_{t}|\Psi_{0:t}\right)\approx \frac{1}{M}\sum_{j=1}^{M}\sum_{i=1}^{N}\omega_{t}^{j,i}\delta \left(X_{t}-X_{t}^{j,i}\right)$$ (22)

where $\Psi_{0:t}$ , *M*, *N* are defined in Eq. 17, $X_{t}$ is defined in Eq. 19, and $X_{t}^{j,i}$ , $\omega_{t}^{j,i}$ are defined in Eq. 21. A smooth variable structure filter (SVSF) is a sliding mode-based predictor-corrector estimator. By having the proper representation of the switching gain, the estimation is converged to be within the boundary of the true state values. This ensures that the estimator is stable and robust to modeling uncertainties and noise. The width of the boundary is referring to the existence subspace that represents the number of uncertainties present in the estimation process. The uncertainties are associated with the inaccuracy of the internal model of the filter and measurement model, that is varies with time. The selection of the width is based on a prior knowledge, since the parameter is not exactly known. During estimation process with proper representation of the boundary, the estimated states are forced to switch back and forth along the true state trajectory by the SVSF gain. However, the switching may cause for chattering effect and can be reduced by introducing the smoothing subspace. For smoothing subspace that is bigger than the existence subspace, the chattering effect is reduced. While for smaller smoothing subspace compared to the existence subspace, the chattering effect is still present (Feng et al., 2011).

## Proposed Data Assimilation Approach

The algorithm of the MIPF–SVSF is the combination of the MIPF and SVSF. The MIPF is functioning to handle the nonlinearity and missing data problem, while the SVSF is used to deal the noise problem. The algorithm of the MIPF–SVSF method is presented as follows:

### Initialization

$${\hat{x}}_{0},CovP_{0|0},CovR,CovQ,\omega_{0},e_{0}$$ (23)

where ${\hat{x}}_{0}$ is the initial state, $CovP_{0|0}$ is the initial prediction error covariance, CovQ is the system noise covariance matrix, CovR is the measurement noise covariance matrix, and $\omega_{0}$ is the initial weight for particle filtering and $e_{0}$ is the initial error difference between the observation and the estimated measurement.

### Prediction

Draw random imputation/observation from proposal function (as in MIPF) from the previously available measurement

$${\text{Ξ}}_{t}^{j}∼\phi \left({\text{Ξ}}_{t}|{\text{Γ}}_{0:t}\right)=\sum_{i=1}^{N}{\tilde{\omega }}_{t}^{i}p\left({\text{Ξ}}_{t}|{\tilde{x}}_{t}^{i}\right)\;for\;i=1,\ldots ,N\;and\;j=1,\ldots M$$ (24)

$$W_{t}^{j}=\left\{{\text{Γ}}_{t},{\text{Ξ}}_{t}^{j}\right\}$$ (25)

where $\Xi_{t}$ is the missing observation at time *t*, $\Gamma_{0:t}$ represent all available observation from time 0 to time *t*, ${\tilde{x}}_{t}^{i}$ is the system state during available observation, ${\tilde{\omega }}_{t}$ is the weight for particle filtering during available observation, $W_{t}^{j}$ is the overall measurement that includes the available observation and new imputation, *N* is the number of particles, and *M* is the number of imputations.

- Prediction in SVSF involves producing the one step state estimation and the covariance *P* for particles generation

$${\hat{x}}_{t|t-1}=f\left({\hat{x}}_{t-1|t-1},u_{t-1|t-1}\right)$$ (26)

$$A_{t}=\frac{\partial f}{\partial x}{|}_{{\hat{x}}_{t|t-1}u_{t-1|t-1},}$$ (27)

$$CovP_{t|t-1}=A_{t}.CovP_{t-1|t-1}.A_{t}^{T}+CovQ_{t|t-1}$$ (28)

$${\hat{\zeta }}_{t|t-1}=g\left({\hat{x}}_{t|t-1}\right)$$ (29)

where $u_{t-1|t-1}$ is the input, ${\hat{x}}_{t-1|t-1}$ is the previous system state, ${\hat{x}}_{t|t-1}$ is the one step estimated state, ${\hat{\zeta }}_{t|t-1}$ is the estimated measurement, $CovP_{t-1|t-1}$ is the previous prediction error covariance, $CovP_{t|t-1}$ is the new prediction error covariance, $CovQ_{t|t-1}$ is the system noise covariance, and *A* is the linearized system model by using Jacobian as represented in Eq. 27.

- Updating in SVSF involve updating the state estimation and covariance *P* for particles generation

$$e_{W,t|t-1}^{j}={\text{W}}_{t}^{j}-{\hat{\zeta }}_{t|t-1}$$ (30)

$$B_{t}=\frac{\partial g}{\partial x}{|}_{{\hat{x}}_{t|t-1},}$$ (31)

$$K_{svsf,t}^{j}=B_{t}^{-1}.diag\left[\left(\left|e_{\text{W},t|t-1}^{j}\right|+\vartheta \left|e_{\text{W},t-1|t-1}^{j}\right|\right).sat\left({\text{Ψ}}^{-1}e_{\text{W},t|t-1}^{j}\right)\right]\cdot diag{\left(e_{\text{W},t|t-1}^{j}\right)}^{-1}$$ (32)

$${\hat{x}}_{t|t}^{j}={\hat{x}}_{t|t-1}+K_{svsf,t}^{j}e_{\text{W},t|t-1}^{j}$$ (33)

$$CovP_{t|t}^{j}=\left(I-K_{svsf,t}^{j}B\right)CovP_{t|t-1}^{j}\left(I-K_{svsf,t}^{j}B\right)T+K_{svsf,t}^{j}CovR_{t|t}{\left(K_{svsf,t}^{j}\right)}^{T}$$ (34)

where ϑ is the convergence rate, $e_{\text{W},t|t-1}^{j}$ is the error difference between the overall observation (that includes the new observation that replaces the missing observation and the available observation) and the estimated measurement, $B_{t}$ is the linearized measurement model by using Jacobian as represented in Eq. 31, Ψ is the smoothing boundary layer vector, and $\Psi^{-1}$ is the diagonal matrix constructed from $n_{k}$ number of Ψ represented as

$${\text{Ψ}}^{-1}=\left[\begin{array}{ccc} \frac{1}{{\text{Ψ}}_{1}} & 0 & 0\\ 0 & \ddots & 0\\ 0 & 0 & \frac{1}{{\text{Ψ}}_{n_{k}}} \end{array}\right]$$ (35)

the saturation function, $sat\left(\Psi^{-1}e_{\text{W},t|t-1}^{j}\right)$ is defined by:

$$sat\left({\text{Ψ}}^{-1}e_{\text{W},t|t-1}^{j}\right)=\left\{\begin{array}{c} 1\\ e_{{\text{W}}_{\text{i}},t|t-1}^{j}/{\text{Ψ}}_{\text{i}}\\ -1 \end{array}\;\begin{array}{c} ,e_{{\text{W}}_{\text{i}},t|t-1}^{j}/{\text{Ψ}}_{\text{i}}\geq 1\\ ,-1<e_{{\text{W}}_{\text{i}},t|t-1}^{j}/{\text{Ψ}}_{\text{i}}<1\;\\ e_{{\text{W}}_{\text{i}},t|t-1}^{j}/{\text{Ψ}}_{\text{i}}\leq -1 \end{array}\right\}\;for\;i=1,\ldots ,n_{k}$$ (36)

- Generate *N* particles of *M* imputation by using the previously obtained estimated state, ${\hat{x}}_{t|t}^{j}$ and prediction error covariance, $CovP_{t|t}^{j}$

$$x_{t}^{j,i}∼q\left({\hat{x}}_{t|t}^{j},CovP_{t|t}^{j}\right)for\;i=1,\ldots ,N\;and\;j=1,\ldots ,M$$ (37)

### Updating

Determine the important weight of the particles from the previous weight, $\omega_{t-1}^{j,i}$ and the likelihood function that includes the generated particles, $x_{t}^{j,i}$ and the overall measurement $W_{t}^{j}$ .

$${\hat{\omega }}_{t}^{j,i}∼p\left(W_{t}^{j}\text{|}x_{t}^{j,i}\right).\omega_{t-1}^{j,i}$$ (38)

The important weight, ${\hat{\omega }}_{t}^{j,i}$ is normalized with the sum of particles weight is equal to unity $\left(\sum_{i=1}^{N}{\hat{\omega }}_{t}^{j,i}\right)$

$$\omega_{t}^{j,i}=\frac{{\hat{\omega }}_{t}^{j,i}}{\sum_{i=1}^{N}{\hat{\omega }}_{t}^{j,i}}$$ (39)

Next, the effective sample size, $N_{eff}$ from the weights are calculated, in order to measure the degeneracy problem. If the $N_{eff}$ is smaller than the threshold (e.g.: $N_{eff}$ <0.5xN), severe degeneracy problem might occur. So, the particles with small weights are eliminated and concentrate on the particles with large weights. The eliminated particles are replaced with the new set of particles from resampling process and the weight is represented as in Eq. 41.

$$N_{eff}=\frac{1}{\sum_{i=1}^{N}{\left({\hat{\omega }}_{t}^{j,i}\right)}^{2}}$$ (40)

$$\omega_{t}^{j,i}=1/N$$ (41)

where ${\hat{\omega }}_{t}^{j,i}$ is defined in Eq. 38, $\omega_{t}^{j,i}$ is the new weight if the degeneracy problem occur, *N* is defined in Eq. 14. By using the generated particles and their associated weights, the estimated states are represented as follows

$${\hat{x}}_{t|t}=\frac{1}{M}\sum_{j=1}^{M}\sum_{i=1}^{N}\omega_{t}^{j,i}x_{t}^{j,i}$$ (42)

where $\omega_{t}^{j,i}$ is defined in Eqs 39, 41, $x_{t}^{j,i}$ is defined in Eq. 37. For the purpose of next iteration in estimation, the updated measurement estimate, ${\hat{\zeta }}_{t|t}$ is determined and used to produce the updated measurement error, $e_{\text{W},t|t}^{j}$ as shown in Eqs 43, 44 respectively.

$${\hat{\zeta }}_{t|t}=g\left({\hat{x}}_{t|t}\right)$$ (43)

$$e_{\text{W},t|t}^{j}=W_{t}^{j}-{\hat{\zeta }}_{t|t}$$ (44)

where $W_{t}^{j}$ is defined in Eq. 25, ${\hat{x}}_{t|t}$ is defined in Eq. 42.

## Convergence Analysis

In order to perform the convergence analysis, the state-space model of the system and observation, and the MIPF–SVSF are reformulated into probability representation.

### Probability Space Formulation

Let (Ω,ℱ,P) be a probability space where $F=\text{Β}\left({\mathbb{R}}^{n_{x}}\right)$ is the Borel set of ${\mathbb{R}}^{n_{x}}$ , the Borel set is the standard set of all possible probability events on ${\mathbb{R}}^{n_{x}}$ . Two types of vector-valued stochastic process namely system state, $X=\left\{X_{t},t\in \mathbb{N}\right\}$ and observation, $W=\left\{W_{t},t\in \mathbb{N}\right\}$ are involved in this space. The system state, *X* is a Markov process of initial distribution *X*_*o*_∼µ and probability transition kernel, *K*(*x*_*t*_ *|x*_*t-1*_).

$$p\left(X_{t}^{n}\in C_{n}\text{|}X_{t-1}=x_{t-1}\right)=\int_{C_{n}}K\left(x_{t}^{n}|x_{t-1}\right)dx_{t}^{n},C_{n}\in \left({\mathbb{R}}^{n_{x}}\right)\;\text{for}\;n=1:n_{s}$$ (45)

where $X_{t}=\left\{X_{t}^{1},X_{t}^{2},\ldots ,X_{t}^{n_{s}}\right\}$ , $X_{t-1}=\left\{X_{t-1}^{1},X_{t-1}^{2},\ldots ,X_{t-1}^{n_{s}}\right\}$ , *n*_*x*_ is the dimension of the states, *n*_*s*_ is the number of the states. Since there are three states namely flow, stage, and cross section, the current states are represented as $x_{t}=\left\{Q_{t},A_{t},H_{t}\right\}$ , and the states at previous time, $x_{t-1}=\left\{Q_{t-1},A_{t-1},H_{t-1}\right\}$ . The overall observation is represented as $W_{t}=W_{t}^{1},\ldots ,W_{t}^{n_{k}}$ for $1\leq k\leq n_{k}$ of *n*_*k*_ sensors that is independent to each other and have marginal distribution

$$p\left(W_{t}^{k}\in D\text{|}X_{t}=x_{t}\right)=\int_{D}p\left(w_{t}^{k}|x_{t}\right)dw_{t}^{k},D\in B\left({\mathbb{R}}^{n_{w}}\right)$$ (46)

where $w_{t}^{k}$ is the overall observation by *k*th sensor at time *t*, $n_{w}$ is the dimension of the observation and $x_{t}$ is defined in Eq. 45. For missing observation problem, consider the non-response vector-valued stochastic process, $R=\left\{R_{t},t\in \mathbb{N}\right\}$ with $n_{w}$ dimensional vector. The availability of the observation is indicated using $r_{t}^{j}\in \left(0,1\right)$ and introduced the following sets

$$\begin{array}{l} \xi_{t}=\left\{w_{t}^{k}|r_{t}^{k}=0\;for\;0\leq k\leq n_{w}\right\}\\ \gamma_{t}=\left\{w_{t}^{k}|r_{t}^{k}=1\;for\;0\leq k\leq n_{w}\right\} \end{array}$$ (47)

where $\xi_{t}$ is the missing observation at time *t*, $\gamma_{t}$ is the available observation at time *t*, $r_{t}^{k}$ is the indicator for the availability of the observation, $n_{w}$ and $w_{t}^{k}$ are defined in Eq. 46. The probability density of the non-response mechanism that is corresponding to the proposal function to draw imputation as in Eq. 17 is represented as

$$p\left(\xi_{t}\text{|}r_{0:t},\gamma_{0:t}\right)=p\left(\Xi_{t}\in d\xi_{t}|R_{0:t}=r_{0:t},\Psi_{0:t}=\gamma_{0:t}\right)$$ (48)

where $\xi_{t}$ is the missing observation at time *t*, $\gamma_{0:t}$ is the available observation from time 0 to time *t*, and $r_{0:t}$ is the indicator for the availability of the observation from time 0 to time *t*, $\Xi_{t}$ and $\Psi_{0:t}$ are defined in (17).

### Probability Representation for State Estimation

The estimation using MIPF–SVSF is represented by the posterior probability density function that considers both the available observation and the missing observation. The distribution of the overall posterior probability density $\psi_{t}^{n}$ and the probability density of the states during available observation, $\eta_{\beta |\alpha :\beta }^{n}$ are described as follows:

$$\psi_{t}^{n}≜\;p\left(X_{t}^{n}\in dx_{t}^{n}|\Psi_{0:t}=\gamma_{0:t},R_{0:t}=r_{0:t}\right)$$ (49)

$$\eta_{\beta |\alpha :\varsigma }^{n}≜\;p\left(X_{\beta }^{n}\in dx_{\beta }^{n}|\Psi_{\alpha }=\gamma_{\alpha },\ldots ,\Psi_{\varsigma }=\gamma_{\zeta }\right)$$ (50)

where $\Psi_{\alpha }=\gamma_{\alpha },\ldots ,\Psi_{\varsigma }=\gamma_{\varsigma }$ represent the available observation from time α to time ς , β is the time for state during the available observation, $\Psi_{0:t}$ is defined in Eq. 17, $x_{t}$ and $X_{t}$ are defined in Eq. 45, $\gamma_{0:t}$ , $R_{0:t}$ , and $r_{0:t}$ are defined in Eq. 48. For notational convenience, the probability density $\eta_{\beta |\alpha :\varsigma }^{n}$ is written as $\eta_{t|t}^{n}$ . So, the distribution of $\psi_{t}^{n}$ and $\eta_{t|t}^{n}$ are related as follows:

$$\psi_{t}^{n}=\int \eta_{t|t}^{n}p\left(\xi_{t}|\gamma_{0:t}\right)d\xi_{t}$$ (51)

where $\eta_{t|t}^{i}$ is the probability density of the states at time *t*, $\xi_{t},$ and $\gamma_{0:t}$ are defined in Eq. 48, $\psi_{t}^{n}$ is defined in Eq. 49. By applying the standard Bayesian filtering theory, the posterior probability density of the available observation can be expressed as

$$\eta_{t|t}^{n}=\frac{p\left(w_{t}|x_{t}\right)\eta_{t|t-1}^{n}}{\int p\left(w_{t}|x_{t}\right)\eta_{t|t-1}^{n}}$$ (52)

where $\eta_{t|t-1}^{n}$ is the probability density of the states at time *t*-1, $\eta_{t|t}^{n}$ is defined in Eq. 51, $x_{t}$ is defined in Eq. 45, $w_{t}$ is defined in Eq. 46.

By combining Eq. 51 with Eq. 52, the overall posterior probability density can be reformulated into Eq. 53. This equation shows the relationship between the probability density during available observation, $\eta_{t|t-1}^{n}$ and the overall probability density, $\psi_{t}^{n}$ .

$$\psi_{t}^{n}=\int \left(\frac{p\left(w_{t}|x_{t}\right)\eta_{t|t-1}^{n}}{\int p\left(w_{t}|x_{t}\right)\eta_{t|t-1}^{n}n}\right)p\left(\xi_{t}|\gamma_{0:t}\right)d\xi_{t}$$ (53)

Since the missing observation is very much affecting the probability distribution, the additional knowledge of this problem is covered by introducing the empirical distribution, ${\left(\eta_{t|t}^{n}\right)}^{N}$ to (51) as a replacement to the true distribution of the probability density, $\eta_{t|t}^{n}$ . So, the posterior probability density for each state with N particles is represented as

$${\left(\psi^{n}\right)}^{N}=\int {\left(\eta_{t|t}^{n}\right)}^{N}p\left(\xi_{t}|\gamma_{t}\right)d\xi_{t}$$ (54)

where $\gamma_{t}$ is the available observation at time *t*, ${\left(\eta_{t|t}^{n}\right)}^{N}$ is the probability density of each states during available observation, $\xi_{t}$ is defined in Eq. 48. The empirical distribution, ${\left(\eta_{t|t}^{n}\right)}^{N}$ consists of *N* particles that are distributed approximately according to $\eta_{t|t}^{n}$ .

$${\left(\eta_{t|t}^{n}\right)}^{N}=\sum_{i=1}^{N}\omega_{t}^{i}\delta {\left(x_{t}^{n}\right)}^{i}$$ (55)

given

$$\omega_{t}^{i}=\frac{p\left(w_{t}|{\left(x_{t}\right)}^{i}\right)}{\sum_{i=1}^{N}p\left(w_{t}|{\left(x_{t}\right)}^{i}\right)}$$ (56)

${\left(x_{t}\right)}^{i}$ is the set of particles distributed approximately according to $\eta_{t-1|t-1}$ for all state, given by ${\left(x_{t}\right)}^{i}∼K\left(x_{t}|{\left(x_{t-1}\right)}^{i}\right)$ as in the standard bootstrap procedure. Next, the missing observation is incorporated into Eq. 32 by applying naive Monte Carlo approximation with $\xi_{t}^{j}∼p\left(\xi_{t}|\gamma_{t}\right)$ for 0≤j≤M . The posterior probability density of the desired states with *N* and *M* is represented as

$${\left(\psi_{t}^{n}\right)}^{N,M}={\left(\eta_{t|t}^{n}\right)}^{N}\frac{1}{M}\sum_{j=1}^{M}\delta \xi_{t}^{j}$$ (57)

where ${\left(\eta_{t|t}^{n}\right)}^{N}$ is the posterior probability density for the related states with *N* particles, $\xi_{t}^{j}$ is the missing observation at time *t* and *j*th imputation, *N* and *M* are defined in Eq. 17. By referring to the MIPF–SVSF algorithm, the relation between the approximated distributions of the related states $p\left(X_{t}^{n}|\Psi_{0:t}\right)$ and the true density can be analyzed through convergence analysis.

#### Part 1: Prediction Using SVSF

Consider the one step prediction error

$${\tilde{x}}_{t|t-1}=x_{t}-{\hat{x}}_{t|t-1}=f\left(x_{t-1}\right)+\upsilon_{t-1}-f\left({\hat{x}}_{t-1|t-1}\right)$$ (58)

where ${\hat{x}}_{t-1|t-1}$ is the estimate of $x_{t-1}$ at time t−1 with the initial state, ${\hat{x}}_{t|t-1}$ is the one step prediction at time t−1 , $x_{t}$ and $\upsilon_{t-1}$ are defined in Eq. 1.

By using the Taylor series expansion around ${\hat{x}}_{t-1|t-1}$ , $f\left(x_{t-1}\right)$ is linearized as follows

$$f\left(x_{t-1}\right)=f\left({\hat{x}}_{t-1|t-1}\right)+A_{t-1}{\tilde{x}}_{t-1|t-1}+\phi {\tilde{x}}_{t-1|t-1}^{2}$$ (59)

where $A_{t-1}=\frac{\partial f\left(x_{t-1}\right)}{\partial x_{t-1}}{|}_{x_{t-1}={\hat{x}}_{t-1|t-1}}$ , and $\phi {\tilde{x}}_{t-1|t-1}^{2}$ is the high order terms of the Taylor series expansion

$$\phi {\tilde{x}}_{t-1|t-1}^{2}={\text{Λ}}_{t-1}{ℵ}_{1,t-1}L_{t-1}{\tilde{x}}_{t-1|t-1}$$ (60)

where $\Lambda_{t-1}$ is a problem dependent scaling matrix, $L_{t-1}$ is introduce to provide extra degree of freedom to the filter, ${ℵ}_{1,t-1}$ is an unknown time-varying matrix for linearization errors of the dynamical model that satisfies

$${ℵ}_{1,t-1}{ℵ}_{1,t-1}^{T}\leq I$$ (61)

that gives

$${\tilde{x}}_{t|t-1}=\left(A_{t-1}+{\text{Λ}}_{t-1}{ℵ}_{1,t-1}L_{t-1}\right){\tilde{x}}_{t-1|t-1}+\upsilon_{t-1}$$ (62)

where $A_{t-1}$ is defined in Eq. 59, $\upsilon_{t-1}$ is defined in Eq. 1, $\;\Lambda_{t-1}$ is defined in Eq. 60, ${ℵ}_{1,t-1}$ and $L_{t-1}$ are defined in Eq. 60. Applying the Taylor series expansion around ${\hat{x}}_{t|t-1}$ to the $g\left(x_{t-1}\right)$ , the innovation of the filter is represented as

$${\tilde{W}}_{t}={\text{W}}_{t}-g\left({\hat{x}}_{t|t-1}\right)=\left(B_{t}+{\text{Ω}}_{\text{t}}{ℵ}_{2,t}L_{t}\right){\tilde{x}}_{t|t-1}+\varepsilon_{t}$$ (63)

where $B_{t}=\frac{\partial g\left(x_{t}\right)}{\partial x_{t}}{|}_{x_{t}={\hat{x}}_{t|t}}$ , $\Omega_{t}$ is a problem dependent scaling matrix, $L_{t}$ is introduce to provide extra degree of freedom to the filter, ${ℵ}_{2,t}$ is an unknown time-varying matrix for linearization errors of the dynamical model that satisfies ${ℵ}_{2,t}{ℵ}_{2,t}^{T}\leq I$ as in Eq. 61, $\;{\tilde{x}}_{t|t-1}$ is defined in Eq. 62, ${\hat{x}}_{t|t-1}$ is defined in Eq. 59, ${\text{W}}_{t}$ and $\;\varepsilon_{t}$ are defined in Eq. 2. The filtering error is represented as

$${\tilde{x}}_{t|t}=x_{t}-{\hat{x}}_{t|t}=\left(I-K_{svsf,t}\left(B_{t}+{\text{Ω}}_{\text{t}}{ℵ}_{2,t}L_{t}\right)\right){\tilde{x}}_{t|t-1}-K_{svsf,t}\varepsilon_{t}$$ (64)

where $K_{svsf,t}$ is the gain, ${\hat{x}}_{t|t}$ is the estimated state from SVSF, $x_{t}$ is defined in Eq. 45, ${\hat{x}}_{t|t-1}$ is defined in Eq. 59, $B_{t},\;\Omega_{t,\;}{ℵ}_{2,t},\;L_{t}$ are defined in Eq. 63, $\varepsilon_{t}$ is defined in Eq. 2,

Theorem 1 The one step prediction error covariance, $\;CovP_{t|t-1}$ is given by

$$CovP_{t|t-1}=\left(A_{t-1}+{\text{Λ}}_{t-1}{ℵ}_{1,t-1}L_{t-1}\right)CovP_{t-1|t-1}{\left(A_{t-1}+{\text{Λ}}_{t-1}{ℵ}_{1,t-1}L_{t-1}\right)}^{T}+CovQ_{t-1}$$ (65)

where $CovP_{t-1|t-1}$ is the previous prediction covariance, $CovQ_{t-1}$ is defined in Eq. 23, $A_{t-1}$ is defined in Eq. 59, $\upsilon_{t-1}$ is defined in Eq. 1, $\;\Lambda_{t-1}$ is defined in Eq. 60, ${ℵ}_{1,t-1},$ and $L_{t-1}$ are defined in Eq. 60

Theorem 2 The filtering error covariance $CovP_{t|t}$ is given by

$$CovP_{t|t}=\left(I-\left(K_{svsf,t}\left(B_{t}+{\text{Ω}}_{\text{t}}{ℵ}_{2,t}L_{t}\right)\right)\right)CovP_{t|t-1}\times {\left(I-\left(K_{svsf,t}\left(B_{t}+{\text{Ω}}_{\text{t}}{ℵ}_{2,t}L_{t}\right)\right)\right)}^{T}+\left(K_{svsf,t}\left({\text{CovR}}_{\text{t}}\right)K_{svsf,t}^{T}\right)$$ (66)

where $E\left\{\varepsilon_{t}\varepsilon_{t}^{T}\right\}=CovR_{t}$ is defined in Eq. 23, $K_{svsf,t}$ is defined in Eq. 64, $B_{t},\;\Omega_{t,\;}{ℵ}_{2,t},\;L_{t}$ are defined in Eq. 63.

Lemma 1 Given matrices A, H, E, and F with appropriate dimensions such that $FF^{T}\leq I$ . Let X be a symmetric positive definite matrix and γ be an arbitrary positive constant such that $\gamma^{-1}I-EXE^{T}>0$ . Then the following inequality holds

$$\left(A+HFE\right)X{\left(A+HFE\right)}^{T}\leq A\left(X^{-1}-\gamma E^{T}E\right)A^{T}+\gamma^{-1}HH^{T}$$ (67)

Theorem 3: Consider Theorem 1 and Theorem 2 and assume ${ℵ}_{1,t-1}{ℵ}_{1,t-1}^{T}\leq I$ and ${ℵ}_{2,t}{ℵ}_{2,t}^{T}\leq I$ as in Eqs 60, 62 are true. Let $\gamma_{1,t-1},\gamma_{2,t}$ be positive scalars. The upper bound for the one step prediction error covariance matrix and filtering error covariance matrix can be represented by the following Riccati-like difference equations using Lemma 1:

$${\text{Σ}}_{t|t-1}=A_{t-1}{\left({\text{Σ}}_{t-1|t-1}^{-1}-\gamma_{1,t-1}^{-1}L_{t-1}L_{t-1}^{T}\right)}^{-1}A_{t-1}^{T}+\gamma_{1,t-1}^{-1}{\text{Λ}}_{t-1}{\text{Λ}}_{t-1}^{T}+CovQ_{t-1}$$ (68)

$${\text{Σ}}_{t|t}=\left(I-\left(K_{s,t}B_{t}\right)\right){\left(\sum_{t|t-1}^{-1}-\gamma_{2,t}^{-1}L_{t}L_{t}^{T}\right)}^{-1}{\left(I-\left(K_{s,t}B_{t}\right)\right)}^{T}+\gamma_{2,t}^{-1}K_{s,t}\Omega_{\text{t}}\Omega_{\text{t}}^{T}K_{s,t}^{T}+\left(K_{s,t}\left({\text{CovR}}_{\text{t}}\right)K_{s,t}^{T}\right)$$ (69)

where $K_{s,t}$ is the gain like $K_{svsf,t}$ in Eq. 64, $CovQ_{t-1}$ and $CovR_{t}$ are defined in Eq. 23, $A_{t-1}$ is defined in Eq. 59, $\;\Lambda_{t-1}$ is defined in Eq. 60, $L_{t-1}$ is defined in Eq. 60, $B_{t},\;\Omega_{\text{t},\;}{ℵ}_{2,t},\;L_{t}$ are defined in Eq. 63, $\gamma_{1,t-1},\;\gamma_{2,t}\;$ are positive scalars.

With initial condition ${\text{Σ}}_{0|0}=CovP_{0|0}>0$ have positive definite solutions ${\text{Σ}}_{t|t-1}$ and ${\text{Σ}}_{t|t}$ such that for all $0<t<N_{t}$ the following two constraints

$$\gamma_{1,t-1}^{-1}I-L_{t-1}{\text{Σ}}_{t-1|t-1}L_{t-1}^{T}>0$$ (70)

$$\gamma_{2,t}^{-1}I-L_{t}{\text{Σ}}_{t|t-1}L_{t}^{T}>0$$ (71)

are satisfied.

The filter gain for the upper bound $K_{s,t}$ is referring to the $K_{svsf,t}$ in Eq. 32 and minimizes the upper bound so that

$$CovP_{t|t}\leq {\text{Σ}}_{t|t}$$ (72)

Lemma 2 For 0<k<N , suppose that $X=X^{T}>0,S_{k}\left(X\right)=S_{k}^{T}\left(X\right)\in {\mathbb{R}}^{nxn}$ and $H_{k}\left(X\right)=H_{k}^{T}\left(X\right)\in {\mathbb{R}}^{nxn}$ . If

$$S_{k}\left(Y\right)\geq S_{k}\left(X\right),\forall X\leq Y=Y^{T}$$ (73)

and

$$H_{k}\left(Y\right)\geq S_{k}\left(Y\right)$$ (74)

Then the solutions for $M_{k}\;and\;N_{k}$ to the following difference equations are

$$M_{k+1}=S_{k}\left(M_{k}\right),\;N_{k+1}=H_{k}\left(N_{k}\right),\;M_{o}=N_{0}>0$$ (75)

Satisfy. $M_{k}\leq N_{k}$

Proof By referring to Lemma 2, rewrite the error covariance matrices as the function of $CovP_{t|t-1}$ .

$$CovP_{t|t-1}\left(CovP_{t-1|t-1}\right)=\left(A_{t-1}+{\text{Λ}}_{t-1}{ℵ}_{1,t-1}L_{t-1}\right)CovP_{t-1|t-1}{\left(A_{t-1}+{\text{Λ}}_{t-1}{ℵ}_{1,t-1}L_{t-1}\right)}^{T}+CovQ_{t-1}$$ (76)

$$CovP_{t|t}\left(CovP_{t|t-1}\right)=\left(I-\left(K_{svsf,t}\left(B_{t}+{\text{Ω}}_{\text{t}}{ℵ}_{2,t}L_{t}\right)\right)\right).CovP_{t|t-1}\;\times {\left(I-\left(K_{svsf,t}\left(B_{t}+{\text{Ω}}_{\text{t}}{ℵ}_{2,t}L_{t}\right)\right)\right)}^{T}+\left(K_{svsf,t}\left({\text{CovR}}_{\text{t}}\right)K_{svsf,t}^{T}\right)$$ (77)

where $CovP_{t-1|t-1}$ is defined in Eq. 65, $\;CovQ_{t-1}$ , $\;CovR_{t}$ are defined in Eq. 23, $A_{t-1}$ is defined in Eq. 59, $\upsilon_{t-1}$ is defined in Eq. 1), $\Lambda_{t-1}$ , ${ℵ}_{1,t-1}$ , and $L_{t-1}$ are defined in Eq. 60, $\;K_{svsf,t}$ is defined in Eq. 56, $B_{t},\;\Omega_{\text{t},\;}{ℵ}_{2,t},\;L_{t}$ are defined in Eq. 63.

Next, rewrite the upper bound obtained in Eq. 67 and Eq. 68 as the function of ${\text{Σ}}_{t-1|t-1}$ and ${\text{Σ}}_{t|t-1}$

$${\text{Σ}}_{t|t-1}\left({\text{Σ}}_{t-1|t-1}\right)=A_{t-1}{\left({\text{Σ}}_{t-1|t-1}^{-1}-\gamma_{1,t-1}^{-1}L_{t-1}L_{t-1}^{T}\right)}^{-1}A_{t-1}^{T}+\gamma_{1,t-1}^{-1}{\text{Λ}}_{t-1}{\text{Λ}}_{t-1}^{T}+CovQ_{t-1}$$ (78)

$${\text{Σ}}_{t|t}\left({\text{Σ}}_{t|t-1}\right)=\left(I-\left(K_{s,t}B_{t}\right)\right){\left({\text{Σ}}_{t|t-1}^{-1}-\gamma_{2,t}^{-1}L_{t}L_{t}^{T}\right)}^{-1}{\left(I-\left(K_{s,t}B_{t}\right)\right)}^{T}+\gamma_{2,t}^{-1}K_{s,t}\Omega_{\text{t}}\Omega_{\text{t}}^{T}K_{s,t}^{T}+\left(K_{s,t}\left({\text{CovR}}_{\text{t}}\right)K_{s,t}^{T}\right)$$ (79)

where $K_{s,t}$ is defined in Eq. 61, $CovQ_{t-1}$ and $CovR_{t}$ are defined in Eq. 23, $A_{t-1}$ is defined in Eq. 59, $\;\Lambda_{t-1}$ , $L_{t-1}$ is defined in Eq. 60, $B_{t},\;\Omega_{\text{t},\;}{ℵ}_{2,t},\;L_{t}$ are defined in Eq. 63, $\gamma_{1,t-1},\gamma_{2,t}$ are positive scalars.

Consider Eqs 75–78, the error covariance matrices and the upper bound satisfy condition Eq. 72, 73 in Lemma 2.

$$CovP_{t|t}\left(CovP_{t|t-1}\right)\geq CovP_{t|t-1}\left(CovP_{t-1|t-1}\right)$$ (80)

$$\sum_{t|t}\left({\text{Σ}}_{t|t-1}\right)\geq CovP_{t|t}\left(CovP_{t|t-1}\right)$$ (81)

which gives $CovP_{t|t}\leq {\text{Σ}}_{t|t}$ .

#### Part 2: Prediction Using MIPF

Consider: if ${\left(\mu_{N}\right)}_{N=1}^{\infty }$ is a sequence of random probability measures, then $\mu_{N}$ converges to $\mu \in B\left({\mathbb{R}}^{n_{x}}\right)$ if for any continuous bounded function $\varphi \in B\left({\mathbb{R}}^{n_{x}}\right)$

$$\underset{N\to \infty }{\lim}E\left[{\left(\left(\mu_{N},\varphi \right)-\left(\mu ,\varphi \right)\right)}^{2}\right]=0$$

Assumption The likelihood function $p\left(W_{t}\text{|}X_{t}\right)$ is a bounded function in argument and represented as π<∞ .

Lemma 3 For any $\varphi \in B\left({\mathbb{R}}^{n_{x}}\right)$ with random variables ${\left\{x_{t}^{i}\right\}}_{i=1}^{N}$ obtained from states, ${\hat{x}}_{t|t}$ and prediction error covariance matrices, $CovP_{t|t}$ from SVSF as in Eq. 37.

$$\begin{array}{l} E{\left|\left({\left(\eta_{t-1|t-1}^{n}\right)}^{N},\varphi \right)-\left(\eta_{t-1|t-1}^{n},\varphi \right)\right|}^{4}=\frac{1}{N^{4}}E{\left[\sum_{i=1}^{N}\left(f\left(x_{t}^{i}\right)-E\left(f\left(x_{t}^{i}\right)\right)\right)\right]}^{4}\;\leq \frac{2}{N^{2}}E{\left[f\left(x_{t}^{i}\right)-E\left(f\left(x_{t}^{i}\right)\right)\right]}^{4}\\ \leq C_{t-1|t-1}\frac{{\left\|\varphi \right\|}^{2}}{N}\;\;for\;n=1:n_{s} \end{array}$$ (82)

Then, for any $\varphi \in B\left({\mathbb{R}}^{n_{x}}\right)$

$$E{\left|\left({\left(\eta_{t|t-1}^{n}\right)}^{N},\varphi \right)-\left(\eta_{t|t-1}^{n},\varphi \right)\right|}^{2}=C_{t|t-1}^{n}\frac{{\left\|\varphi \right\|}^{2}}{N}$$ (83)

where $\eta_{t-1|t-1}^{n}$ is the probability density of the random variables, $\eta_{t|t-1}^{n}$ is the probability density of the one step state estimate, $C_{t|t-1}^{n}$ and $C_{t-1|t-1}^{n}$ are the constant, $x_{t}^{i}$ is the particles obtained, and $n_{s}$ is the number of the states; N is described in Eq. 37.

Proof Consider

$$\begin{array}{l} \left|\left({\left(\eta_{t|t-1}^{n}\right)}^{N},\varphi \right)-\left(\eta_{t|t-1}^{n},\varphi \right)\right|\\ \leq \left|\left({\left(\eta_{t|t-1}^{n}\right)}^{N},\varphi \right)-\left({\left(\eta_{t-1|t-1}^{n}\right)}^{N},K\varphi \right)\right|+\left|\left({\left(\eta_{t-1|t-1}^{n}\right)}^{N},K\varphi \right)-\left(\eta_{t-1|t-1}^{n},K\varphi \right)\right| \end{array}$$ (84)

Let $G_{t-1}$ be the σ−field generated by ${\left\{x_{t-1}^{i}\right\}}_{i=1}^{N}$ , then

$$E\left[\left(\eta_{t|t-1}^{N},\varphi \right)|G_{t-1}\right]=\left(\eta_{t-1|t-1}^{N},K\varphi \right)$$ (85)

and, as Kφ≤φ

$$E\left[{\left(\left({\left(\eta_{t|t-1}^{n}\right)}^{N},\varphi \right)-E\left(\left({\left(\eta_{t|t-1}^{n}\right)}^{N},K\varphi \right)\right)|G_{t-1}\right)}^{2}|G_{t-1}\right]\leq \frac{{\left\|\varphi \right\|}^{2}}{N}$$ (86)

Using Minkowski’s inequality

$$E{\left[{\left(\left({\left(\eta_{t|t-1}^{n}\right)}^{N},\varphi \right)-\left(\eta_{t|t-1}^{n},\varphi \right)\right)}^{2}\right]}^{\frac{1}{2}}\leq \sqrt{C_{t-1|t-1}^{n}}\frac{\varphi }{\sqrt{N}}+\frac{\varphi }{\sqrt{N}}\leq C_{t|t-1}^{n}\frac{\left\|\varphi \right\|}{\sqrt{N}}$$ (87)

where $C_{t|t-1}^{n}={\left(\sqrt{C_{t-1|t-1}^{n}}+1\right)}^{2}$ .

#### Part 3: Updating Using MIPF

Lemma 4 For any $\varphi \in B\left({\mathbb{R}}^{n_{x}}\right)$

$$E{\left[\left({\left(\eta_{t|t-1}^{n}\right)}^{N},\varphi \right)-\left(\eta_{t|t-1}^{n},\varphi \right)\right]}^{2}\leq C_{t|t-1}^{n}\frac{{\left\|\varphi \right\|}^{2}}{N}\;$$ (88)

$where\;C_{t|t-1}^{n}={\left(\sqrt{C_{t-1|t-1}^{n}}+1\right)}^{2}$

Then for any $\varphi \in B\left({\mathbb{R}}^{n_{x}}\right)$

$$E{\left[\left({\left({\tilde{\eta }}_{t|t}^{n}\right)}^{N},\varphi \right)-\left(\eta_{t|t}^{n},\varphi \right)\right]}^{2}={\tilde{C}}_{t|t-1}^{n}\frac{{\left\|\varphi \right\|}^{2}}{N}$$ (89)

where $\eta_{t|t-1}^{n}$ is the probability density of the one step state estimate, ${\tilde{\eta }}_{t|t}^{n}$ is the probability density of the state without consideration of missing data, $C_{t|t-1}^{n}$ and ${\tilde{C}}_{t|t-1}^{n}$ are the constant, N is described in Eq. 37.

Proof: Consider

$$\left({\left({\tilde{\eta }}_{t|t}^{n}\right)}^{N},\varphi \right)-\left(\eta_{t|t}^{n},\varphi \right)=\frac{\left({\left(\eta_{t|t-1}^{n}\right)}^{N},\pi \varphi \right)}{\left({\left(\eta_{t|t-1}^{n}\right)}^{N},\pi \right)}-\frac{\left(\eta_{t|t-1}^{n},\pi \varphi \right)}{\left(\eta_{t|t-1}^{n},\pi \right)}$$ (90)

For the first part of Eq. 90

$$\left|\frac{\left({\left(\eta_{t|t-1}^{n}\right)}^{N},\pi \varphi \right)}{\left({\left(\eta_{t|t-1}^{n}\right)}^{N},\pi \right)}-\frac{\left({\left(\eta_{t|t-1}^{n}\right)}^{N},\pi \varphi \right)}{\left(\eta_{t|t-1}^{n},\pi \right)}\right|\leq \frac{\varphi }{\left(\eta_{t|t-1}^{n},\pi \right)}\left|\left(\eta_{t|t-1}^{n},\pi \right)-\left({\left(\eta_{t|t-1}^{n}\right)}^{N},\pi \right)\right|$$ (91)

Using Minkowski’s inequality

$$E{\left[{\left(\left({\left({\tilde{\eta }}_{t|t}^{n}\right)}^{N},\varphi \right)-\left(\eta_{t|t}^{n},\varphi \right)\right)}^{2}\right]}^{\frac{1}{2}}\leq \frac{\left\|\varphi \right\|}{\left({\left(\eta_{t|t-1}^{n}\right)}^{N},\pi \right)}E{\left[{\left(\left(\eta_{t|t-1}^{n},\pi \varphi \right)-\left({\left(\eta_{t|t-1}^{n}\right)}^{N},\pi \right)\right)}^{2}\right]}^{\frac{1}{2}}+\frac{E{\left[{\left(\left({\left(\eta_{t|t-1}^{n}\right)}^{N},\pi \varphi \right)-\left(\eta_{t|t-1}^{n},\pi \varphi \right)\right)}^{2}\right]}^{\frac{1}{2}}}{\left({\left(\eta_{t|t-1}^{n}\right)}^{N},\pi \right)}\leq \frac{2\sqrt{C_{t|t-1}^{n}}\left\|\pi \right\|}{\left({\left(\eta_{t|t-1}^{n}\right)}^{N},\pi \right)}\frac{\left\|\varphi \right\|}{\sqrt{N}}$$ (92)

#### Part 4: Updating With New Imputation Using MIPF

Lemma 5 For any $\varphi \in B\left({\mathbb{R}}^{n_{x}}\right)$

$$E{\left[\left({\left({\tilde{\eta }}_{t|t}^{n}\right)}^{N},\varphi \right)-\left(\eta_{t|t}^{n},\varphi \right)\right]}^{2}\leq {\tilde{C}}_{t|t-1}^{n}\frac{{\left\|\varphi \right\|}^{2}}{N}\;$$ (93)

$\text{where}\;{\tilde{C}}_{t|t-1}^{n}=\;{\left(\frac{2\sqrt{C_{t|t-1}^{n}}\pi }{\left({\left(\eta_{t|t-1}^{n}\right)}^{N},\pi \right)}\right)}^{2}$ . Then, for any $\varphi \in B\left({\mathbb{R}}^{n_{x}}\right)$ that includes new imputation $\varrho \in B\left({\mathbb{R}}^{n_{z}}\right)$

$$E{\left[\left({\left({\tilde{\text{Ψ}}}_{t|t}^{n}\right)}^{N,M},\varphi \right)-\left({\text{Ψ}}_{t|t}^{n},\varphi \right)\right]}^{2}={\tilde{f}}_{t|t}^{n}\frac{{\left\|\varphi \right\|}^{2}}{MN}$$ (94)

where ${\tilde{\eta }}_{t|t}^{n}$ is the probability density of the state without the consideration of missing data, ${\tilde{\Psi }}_{t|t}^{n}$ is the probability density of the state with the consideration of missing data, and ${\tilde{C}}_{t|t-1}^{n}$ and ${\tilde{f}}_{t|t}^{n}$ are the constant; M and N are described in Eq. 37.

Proof: Consider

$$\left({\left({\tilde{\eta }}_{t|t}^{n}\right)}^{N}H_{t}^{M},\varphi \right)-\left(\eta_{t|t}^{n}H_{t},\varphi \right)=\frac{\left({\left(\eta_{t|t-1}^{n}\right)}^{N}H_{t}^{M},\varrho \pi \varphi \right)}{\left({\left(\eta_{t|t-1}^{n}\right)}^{N}H_{t}^{M},\varrho \pi \right)}-\frac{\left({\left(\eta_{t|t-1}^{n}\right)}^{N}H_{t}^{M},\varrho \pi \varphi \right)}{\left(\eta_{t|t-1}^{n}H_{t},\varrho \pi \right)}+\frac{\left({\left(\eta_{t|t-1}^{n}\right)}^{N}H_{t}^{M},\varrho \pi \varphi \right)}{\left(\eta_{t|t-1}^{n}H_{t},\varrho \pi \right)}-\frac{\left(\eta_{t|t-1}^{n}H_{t},\varrho \pi \varphi \right)}{\left(\eta_{t|t-1}^{n}H_{t},\varrho \pi \right)}\;$$ (95)

For the first part of Eq. 95

$$\left|\frac{\left({\left(\eta_{t|t-1}^{n}\right)}^{N}H_{t}^{M},\varrho \pi \varphi \right)}{\left({\left(\eta_{t|t-1}^{n}\right)}^{N}H_{t}^{M},\varrho \pi \right)}-\frac{\left({\left(\eta_{t|t-1}^{n}\right)}^{N}H_{t}^{M},\varrho \pi \varphi \right)}{\left(\eta_{t|t-1}^{n}H_{t},\varrho \pi \right)}\right|\leq \frac{\left\|\varphi \right\|}{\left(\eta_{t|t-1}^{n}H_{t},\varrho \pi \right)}\left|\left(\eta_{t|t-1}^{n}H_{t},\varrho \pi \right)-\left({\left(\eta_{t|t-1}^{n}\right)}^{N}H_{t}^{M},\varrho \pi \right)\right|$$ (96)

For the second part of Eq. 94 that refer to Eq. 89

$${\left|\frac{\left({\left(\eta_{t|t-1}^{n}\right)}^{N}H_{t}^{M},\varrho \pi \varphi \right)}{\left(\eta_{t|t-1}^{n}H_{t},\varrho \pi \right)}-\frac{\left(\eta_{t|t-1}^{n}H_{t},\varrho \pi \varphi \right)}{\left(\eta_{t|t-1}^{n}H_{t},\varrho \pi \right)}\right|}^{2}={\tilde{f}}_{t|t-1}^{n}\frac{{\left\|\varphi \right\|}^{2}}{MN}$$ (97)

Using Minkowski’s inequality

$$E{\left[{\left(\left({\left({\tilde{\eta }}_{t|t}^{n}\right)}^{N}H_{t}^{M},\varphi \right)-\left(\eta_{t|t}^{n}H_{t},\varphi \right)\right)}^{2}\right]}^{\frac{1}{2}}\leq \frac{2\sqrt{{\tilde{f}}_{t|t-1}^{n}}\varrho \pi }{\left(\eta_{t|t-1}^{n}H_{t},\varrho \pi \right)}\;\frac{\left\|\varphi \right\|}{\sqrt{MN}}$$ (98)

#### Part 5: Resampling Using MIPF

Lemma 6 For any $\varphi \in B\left({\mathbb{R}}^{n_{x}}\right)$ and $\varrho \in B\left({\mathbb{R}}^{n_{z}}\right)$

$$E{\left|\left({\left({\tilde{\text{Ψ}}}_{t|t}^{n}\right)}^{N,M},\varphi \right)-\left({\text{Ψ}}_{t|t}^{n},\varphi \right)\right|}^{2}\leq {\tilde{f}}_{t|t}^{n}\frac{{\left\|\varphi \right\|}^{2}}{MN}with\;{\tilde{f}}_{t|t}^{n}={\left(\frac{2\sqrt{{\tilde{f}}_{t|t-1}^{n}}\varrho \pi }{\left(\eta_{t|t-1}^{n}H_{t},\varrho \pi \right)}\right)}^{2}$$ (99)

There exists a constant $f_{t|t}^{n}$ , such that for any $\varphi \in B\left({\mathbb{R}}^{n_{x}}\right)$ and $\varrho \in B\left({\mathbb{R}}^{n_{z}}\right)$

$$E{\left|\left({\left({\text{Ψ}}_{t|t}^{n}\right)}^{N,M},\varphi \right)-\left({\text{Ψ}}_{t|t}^{n},\varphi \right)\right|}^{2}=f_{t|t}^{n}\frac{{\left\|\varphi \right\|}^{2}}{MN}$$ (100)

where ${\tilde{\Psi }}_{t|t}^{n}$ is the probability density of the state with the consideration of missing data, $\Psi_{t|t}^{n}$ is the posterior probability density of the states, and ${\tilde{C}}_{t|t-1}^{n}$ and ${\tilde{f}}_{t|t}^{n}$ are the constant; M and N are described in Eq. 37.

Proof Consider the following

$$\left({\left({\text{Ψ}}_{t|t}^{n}\right)}^{N,M},\varphi \right)-\left({\text{Ψ}}_{t|t}^{n},\varphi \right)=\left({\left({\text{Ψ}}_{t|t}^{n}\right)}^{N,M},\varphi \right)-\left({\left({\tilde{\text{Ψ}}}_{t|t}^{n}\right)}^{N,M},\varphi \right)+\left({\left({\tilde{\text{Ψ}}}_{t|t}^{n}\right)}^{N,M},\varphi \right)-\left({\text{Ψ}}_{t|t}^{n},\varphi \right)$$ (101)

By using Minkowski’s inequality

$$\begin{array}{l} E{\left[{\left(\left({\left({\text{Ψ}}_{t|t}^{n}\right)}^{N,M},\varphi \right)-\left({\text{Ψ}}_{t|t}^{n},\varphi \right)\right)}^{2}\right]}^{\frac{1}{2}}\\ \leq E{\left[{\left(\left({\left({\text{Ψ}}_{t|t}^{n}\right)}^{N,M},\varphi \right)-\left({\left({\tilde{\text{Ψ}}}_{t|t}^{n}\right)}^{N,M},\varphi \right)\right)}^{2}\right]}^{\frac{1}{2}}\;+E{\left[{\left(\left({\left({\tilde{\text{Ψ}}}_{t|t}^{n}\right)}^{N,M},\varphi \right)-\left({\text{Ψ}}_{t|t}^{n},\varphi \right)\right)}^{2}\right]}^{\frac{1}{2}} \end{array}$$ (102)

Let $F_{t-1}$ be the σ−field generated by ${\left\{{\tilde{x}}_{t-1}^{i}\right\}}_{i=1}^{N}$ , then

$$E\left[\left({\left({\text{Ψ}}_{t|t}^{n}\right)}^{N,M},\varphi \right)|F_{t-1}\right]=\left({\left({\tilde{\text{Ψ}}}_{t|t}^{n}\right)}^{N,M},\varphi \right)$$ (103)

and

$$E\left[{\left(\left({\left({\text{Ψ}}_{t|t}^{n}\right)}^{N,M},\varphi \right)-\left({\left({\tilde{\text{Ψ}}}_{t|t}^{n}\right)}^{N,M},\varphi \right)\right)}^{2}|F_{t-1}\right]=\frac{F}{MN}{\left\|\varphi \right\|}^{2}$$ (104)

That gives

$$E{\left[{\left(\left({\left({\text{Ψ}}_{t|t}^{n}\right)}^{N,M},\varphi \right)-\left({\text{Ψ}}_{t|t}^{n},\varphi \right)\right)}^{2}\right]}^{\frac{1}{2}}\leq \frac{\sqrt{F}+{\tilde{f}}_{t|t}^{n}}{\sqrt{MN}}\left\|\varphi \right\|$$ (105)

Theorem 4For all t>0 there exist $f_{t|t}^{n}$ that is independent of N but being influenced by M for any bounded function, $\;\varphi \in B\left({\mathbb{R}}^{n_{x}}\right)$

$$E\left[{\left(\left({\left({\text{Ψ}}_{t|t}^{n}\right)}^{N,M},\varphi \right)-\left({\text{Ψ}}_{t|t}^{n},\varphi \right)\right)}^{2}\right]\leq f_{t|t}^{n}\frac{{\left\|\varphi \right\|}^{2}}{MN}\;with\;f_{t|t}^{n}={\left(\sqrt{F}+{\tilde{f}}_{t|t}^{n}\;\right)}^{2}$$ (106)

$$E\left[{\left(\left({\left({\text{Ψ}}_{t|t}^{n}\right)}^{N,M},\varphi \right)-\left({\text{Ψ}}_{t|t}^{n},\varphi \right)\right)}^{2}\right]\leq \frac{1}{M^{2}N^{2}}E{\left[\sum_{j=1}^{M}\sum_{i=1}^{N}\left(f\left(x_{t}^{j,i}\right)-E\left(f\left(x_{t}^{j,i}\right)\right)\right)\right]}^{2}$$ (107)

where $\Psi_{t|t}^{n}$ is the posterior probability density of the states, $f_{t|t}^{n}$ is the constant, M and N are described in Eq. 37, $x_{t}^{j,i}$ is particles generated as in Eq. 37. The number of imputations, M for missing observation data is related to the number of particles, N since at each imputation contain several particles for estimation. To ensure the accuracy of estimation, the number of imputation M and the number of particles N are considered based on the dimension of the missing observation data, $n_{z}$ and dimension of the states, $\;n_{x}$ respectively. Besides that, the number of M and N also are influenced by the Gaussian noise of the measurement error that affects the likelihood function as in Eq. 16. Large error difference in the likelihood function requires high number of M and N to help for the convergence of the filter to the true state. By having proposed states and error covariance matrices to generate the particles as in Eq. 37 that are determined through considering the error difference of the observation and estimated measurement, the distribution of the particles can be limit to the relevant area only. Therefore, the number of M and N needed by this method will be less than the particle filtering method that randomly generated particles without reference.

## Results and Discussion

In this section, the estimation of the river flow and stage, and the velocity of the last drifter are presented. Consider the measurements are suffering from the missing data that affect the estimation process. The MIPF–SVSF is proposed to perform the state estimation by applying several numbers of particles and imputations as described in the previous section. The performance of this method is evaluated by finding the root mean square error (RMSE), standard deviation (SD) (Chai and Draxler, 2014), mean absolute error (MAE), and computational time (Liang et al., 2012) between the measured velocity and the estimated velocity. The RMSE, SD, MAE, and computational time of the MIPF–SVSF are compared with the same parameters from forward simulation, EKF during complete data, PF during complete data, and MIPF.

### Description of the Estimation Process

In this research, the measurements from the drifters (Lee et al., 2011) are used in the estimation of the flow, stage, and cross section that are later used to estimate the velocity of the sixth sensor. The estimated sensor velocity is proportional to the velocity of the river flow. During estimation process, the river system is discretized into 60 cells with 5 m interval. The temporal step size is chosen as 1 s. Since the data about the bottom of the river is unavailable, the bed slope is considered as zero.

The estimation is carried out by combining the system and the observation. During no missing data, the estimation is carried out by the PF–SVSF. The MIPF–SVSF is proposed for the state estimation with missing data problem. Consider the observation is suffering from three types of missing data namely missing velocity data, missing position data, and missing combination of velocity and position data at the same time. Whereby each case has 10, 20, and 30% missing data (Ugryumova et al., 2015). During estimation, the missing data is replaced by several number of imputations. The imputations are generated based on the previously available data. For each imputation, the error difference between the estimated measurement by the SVSF, and the observation from the imputation is calculated. This error is bounded by using the smoothing boundary layer vector. The bounded error and the convergence rate are used in producing the SVSF gain to obtain the new state value.

Next, the new state by the SVSF with their covariance are used to generate several particles. The particles and the observation from the imputation is combined to form the likelihood function. From the likelihood function, the important weight of the particles is obtained. The particles and their weight are used to produce estimated state that consists of the flow, stage, and cross-sectional area. The number of estimated states is depending on the number of imputations. The mean of these states is the desired new estimated state by the proposed method. The estimation process is repeated for 400 s with the error difference between the current result and the observation is use in the next estimation cycle. Since the proposed method includes the convergence rate and smoothing boundary layer vector, the error difference between the estimated velocity and the observation is reduced. By reducing the error, the MIPF–SVSF will have less computational time compared to the MIPF.

### Discussion

**FIGURE 1:**
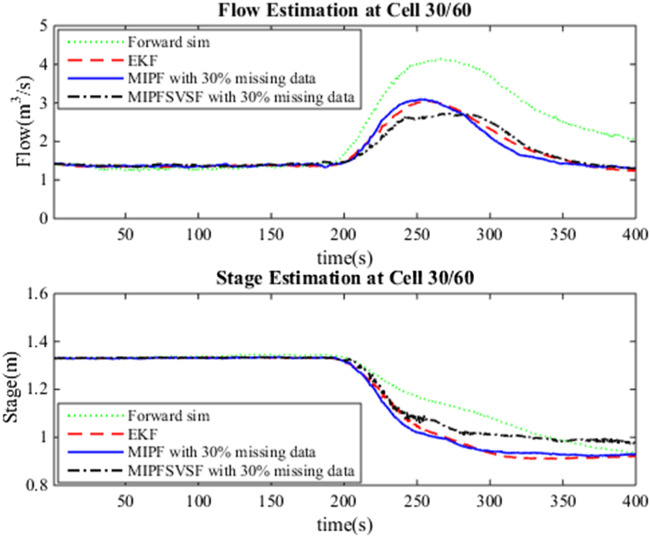
Estimated flow and stage at the 30th cell, forward simulation, EKF during no missing data, MIPF with 30% missing data, and MIPF–SVSF with 30% missing data.

**FIGURE 2:**
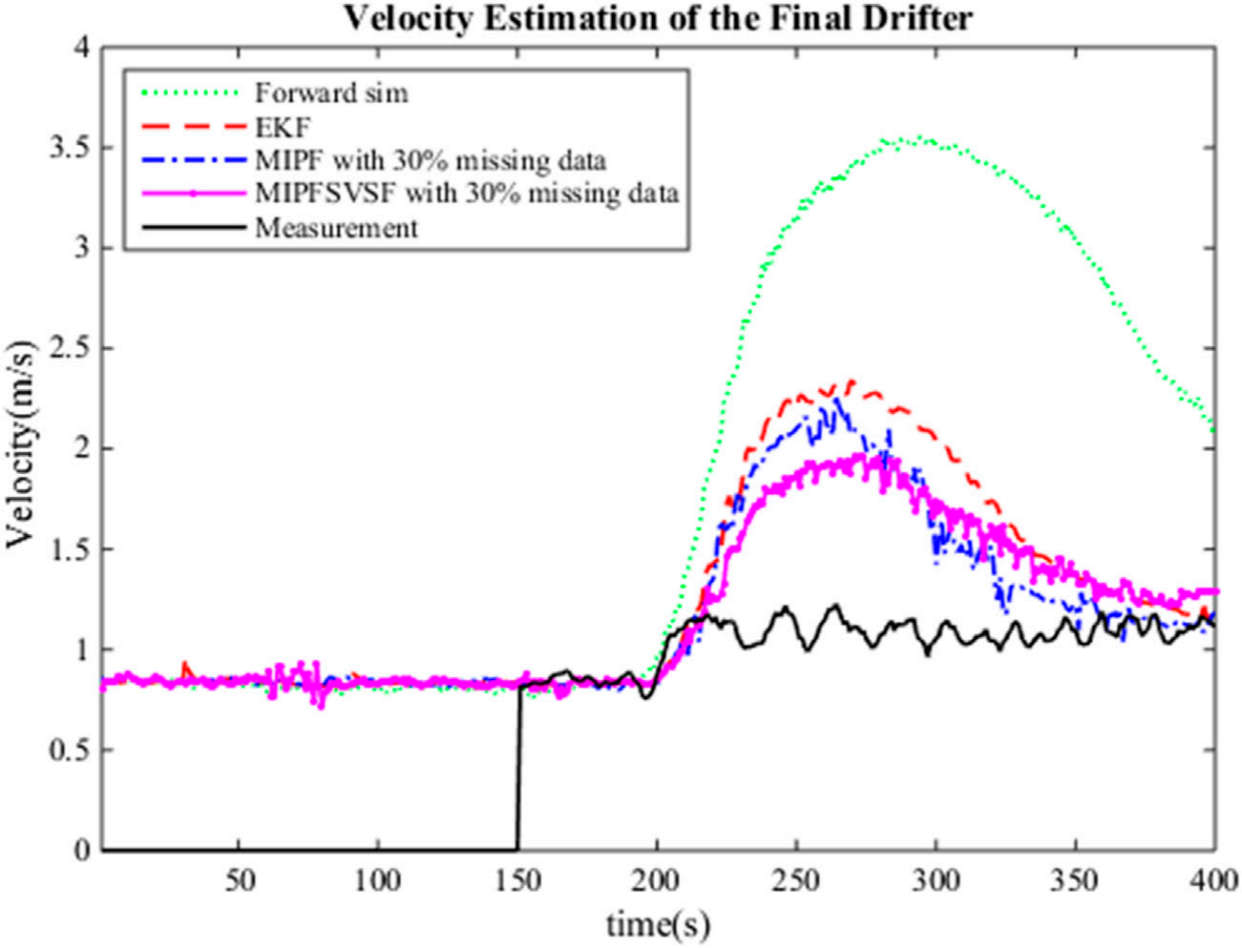
Estimated velocity of the final drifter using forward simulation, EKF during no missing data, MIPF with 30% missing data, and MIPF-SVSF with 30% missing data.

The estimation process by the DA method includes the merging of the system and the observation. Three types of missing data cases are considered in this research as mentioned earlier. For each case, several numbers of imputations, namely 5, 10, 15, and 20 are injected during estimation with 50 particles. The same number of particles are applied for the whole estimation process due to the good estimation result by the PF using these particles during no missing data. Figure 1 shows the estimated flow and stage by forward simulation, the EKF (no missing data), MIPF and MIPF–SVSF for 30% missing velocity data. The overall flow estimation by the MIPF is smaller than the EKF and forward simulation, while the overall estimation by the MIPF–SVSF is smaller than the other methods. Besides that, the MIPF also produce estimated stage that is bigger than the EKF and the forward simulation. However, the estimated stage by the MIPF–SVSF is bigger than the MIPF. The state estimation by the proposed method seems to be reasonable. Therefore, the performance of the method is evaluated by finding the velocity of the final drifter and compared with the measurement. The velocity of the final drifter is obtained by combining the estimated flow and cross-sectional area at the corresponding cell. Figure 2 shows the velocity of the final drifter predicted by the forward simulation, EKF (no missing data), MIPF, and MIPF–SVSF. The figure shows that the proposed method helps the estimation to converge to the desired value. This can be seen by smaller difference between the estimated and the true state, compared to the other methods.

For further analysis and comparison of the performance of the MIPF–SVSF, the RMSE, the SD, the MAE, and the computational time are determined as in Table 1, Table 2 and Table 3. The performance of the MIPF–SVSF is compared with the MIPF and the PF and the EKF from Table 4. Since the proposed method is the upgraded version of the MIPF, the performance of the MIPF is first examined. By considering all missing data cases, the MIPF is able to produce RMSE, SD, and MAE that is close to the estimation result by the PF during complete data. These show that the external input data successfully fill in the missing part. Besides that, the combination of the SVSF with the MIPF improves the estimation result through smaller RMSE, SD, and MAE compared to the MIPF for all missing data cases. This indicates that the SVSF method successfully bound the error difference in the estimation process. The bounded error also helps to reduce the MIPF–SVSF’s computational time compared to the MIPF through the generation of gain that brought the estimation velocity closer to the observation. The results are represented by bold numbers in Tables 1–3. The selection is made based on the smallest value among variables.

**TABLE 1:** Performance of the MIPF and MIPF–SVSF during missing velocity data.

Method	*N*	*M*	Missing velocity data											
			10%				20%				30%			
			RMSE	SD	MAE	Time	RMSE	SD	MAE	Time	RMSE	SD	MAE	Time
MIPF	50	5	0.500	0.437	0.344	59.158	0.500	0.439	0.351	60.564	0.507	0.444	0.351	61.945
		10	**0.502**	**0.434**	**0.345**	**61.363**	0.498	0.437	0.349	63.936	0.508	0.440	0.348	66.452
		15	0.506	0.439	0.348	62.623	0.500	0.441	0.347	67.522	0.507	0.441	0.346	71.077
		20	0.506	0.436	0.351	64.263	**0.503**	**0.438**	**0.346**	**70.894**	**0.502**	**0.438**	**0.346**	**75.787**
MIPF-SVSF	50	5	**0.434**	**0.373**	**0.322**	**6.809**	0.445	0.376	0.338	8.047	0.443	0.371	0.334	8.890
		10	0.437	0.376	0.333	8.233	0.443	0.376	0.333	10.892	0.451	0.379	0.332	13.102
		15	0.435	0.372	0.329	9.716	**0.434**	**0.365**	**0.327**	**13.703**	0.445	0.379	0.333	16.891
		20	0.435	0.375	0.330	11.221	0.438	0.372	0.329	16.682	**0.442**	**0.373**	**0.331**	**20.699**

Bold values represents the smallest value among variables (RMSE SD MAE Time).

**TABLE 2:** The performance of the MIPF and MIPF–SVSF during missing position data.

Method	*N*	*M*	Missing position data											
			10%				20%				30%			
			RMSE	SD	MAE	Time	RMSE	SD	MAE	Time	RMSE	SD	MAE	Time
MIPF	50	5	0.493	0.432	0.347	64.212	0.497	0.432	0.347	66.177	0.501	0.435	0.348	67.914
		10	0.500	0.432	0.346	65.994	0.502	0.434	0.348	70.1.1	0.502	0.433	0.345	73.766
		15	**0.503**	**0.434**	**0.344**	**68.312**	**0.503**	**0.435**	**0.344**	**74.529**	**0.503**	**0.435**	**0.345**	**79.754**
		20	0.502	0.435	0.347	70.403	0.502	0.433	0.348	77.947	0.503	0.434	0.348	84.730
MIPF–SVSF	50	5	**0.439**	**0.362**	**0.338**	**7.162**	0.441	0.373	0.333	8.494	0.441	0.371	0.336	9.155
		10	0.440	0.369	0.335	8.778	**0.438**	**0.372**	**0.333**	**10.972**	0.441	0.376	0.333	13.482
		15	0.442	0.372	0.333	10.239	0.442	0.373	0.333	13.979	**0.440**	**0.366**	**0.336**	**17.643**
		20	0.441	0.382	0.334	11.705	0.440	0.370	0.334	17.022	0.441	0.370	0.336	21.743

Bold values represents the smallest value among variables (RMSE SD MAE Time).

**TABLE 3:** The performance of the MIPF and MIPF-SVSF during missing combination of position and velocity data.

Method	*N*	*M*	Missing combination of velocity and position data											
			10%				20%				30%			
			RMSE	SD	MAE	Time	RMSE	SD	MAE	Time	RMSE	SD	MAE	Time
MIPF	50	5	**0.502**	**0.436**	**0.346**	**65.196**	**0.504**	**0.436**	**0.343**	**66.356**	0.502	0.438	0.351	67.764
		10	0.502	0.435	0.347	67.258	0.502	0.436	0.348	70.214	**0.504**	**0.435**	**0.346**	**73.130**
		15	0.502	0.436	0.345	68.972	0.501	0.433	0.346	75.441	0.501	0.433	0.348	76.950
		20	0.501	0.437	0.349	71.112	0.497	0.435	0.347	77.984	0.499	0.438	0.352	82.081
MIPF–SVSF	50	5	**0.438**	**0.372**	**0.330**	**6.866**	0.443	0.374	0.337	7.960	0.446	0.384	0.334	8.594
		10	0.441	0.378	0.332	8.231	0.441	0.374	0.333	10.821	0.442	0.374	0.335	12.233
		15	0.444	0.377	0.337	9.647	**0.438**	**0.371**	**0.331**	**13.794**	0.448	0.379	0.334	15.838
		20	0.442	0.374	0.336	11.175	0.439	0.376	0.330	16.579	**0.436**	**0.375**	**0.324**	**19.397**

Bold values represents the smallest value among variables (RMSE SD MAE Time).

**TABLE 4:** The performance of the Forward simulation, EKF, and PF during complete data.

Method	*N*	RMSE	SD	MAE	Time
Forward sim	—	1.6778	1.054	1.417	1.511
EKF	—	0.657	0.526	0.480	2.735
PF	50	0.504	0.436	0.339	58.272

By using 50 particles with different number of imputations, the performance of state estimation by MIPF–SVSF during missing velocity data is shown in Table 1. The result shows that the increasing number of imputations will reduce the RMSE, SD, and MAE. However, too many imputations may diverge the estimation from the true value. This can be seen through the sudden increase of RMSE, SD, and MAE after reduction. Besides that, the number of imputations also related to the percentage of missing data. Whereby the higher percentage of missing observation data require higher number of imputations. The same response is shown during the state estimation with missing position data and missing combination of velocity and position data as shown by Tables 2, 3, respectively. The increasing number of imputations reduces the RMSE, SD, and MAE, and the suitable number of imputations is selected before the divergence of the estimation occurred. For different percentages of missing data, the appropriate number of imputations is considered based on the response from RMSE, SD, and MAE.

In this research, the number of particles is fixed to 50 particles throughout the estimation process. Based on the result represented by bold numbers in Tables 1–3, the MIPF–SVSF has less RMSE, SD, MAE, and computational time compared to the MIPF for all missing data cases. The percentage improvement of the proposed method compared to MIPF in terms of RMSE is between 12 and 13.5%, SD is between 14 and 15%, and MAE is between 2 and 7%. While for the computational time, the percentage of reduction is between 73 and 90%. Therefore, the proposed method still can have small RMSE, SD, MAE and computational time under lower number of particles. Since the number of particles and imputations are related, a smaller number of imputations for MIPF–SVSF compared to the MIPF is enough for good estimation performance.

## Conclusion

The MIPF–SVSF is the extension of the MIPF method with the addition of the SVSF. The proposed method introduces several numbers of imputation based on the previously available data to replace the missing data. There are three types of missing data considered in the research, namely, missing velocity data, missing position data, and the missing combination of velocity and position. The convergence analysis of this method shows that the number of particles and imputations depends on the likelihood function that represents error difference between the estimated observation and the imputation data that replaces the missing data. Large error difference requires high number of particles and imputation to converge the estimation to the true state. The SVSF reduces the error difference through the introduction of convergence rate and the smoothing boundary layer vector. These variables are used to generate the particles and their weight and form the estimated state. The performance comparison between MIPF–SVSF and MIPF shows that MIPF–SVSF has better performance than the MIPF in terms of RMSE, SD, MAE and computational time. Besides that, different missing data cases with different percentages of missing data require different combination of particles and imputations. The MIPF–SVSF requires smaller combination of particles and imputation compared to the MIPF for all missing data cases.

## Data Availability

The original contributions presented in the study are included in the article/supplementary material, further inquiries can be directed to the corresponding author.

## References

[B1] Abaza M. Anctil F. Fortin V. Turcotte R. (2014). Sequential Streamflow Assimilation for Short-Term Hydrological Ensemble Forecasting. J. Hydrol. 519, 2692–2706. 10.1016/j.jhydrol.2014.08.038

[B2] Adnan R. M. Yuan X. Kisi O. Anam R. (2017). Improving Accuracy of River Flow Forecasting Using LSSVR with Gravitational Search Algorithm. Adv. Meteorol. 2017. 10.1155/2017/2391621

[B3] Arulampalam M. S. Maskell S. Gordon N. Clapp T. (2002). A Tutorial on Particle Filters for Online Nonlinear/non-Gaussian Bayesian Tracking. IEEE Trans. Signal. Process. 50 (2), 174–188. 10.1109/78.978374

[B4] Aswathy S. Sajikumar N. Mehsa M. (2016). Watershed Modelling Using Control System Concept. Proced. Tech. 24, 39–46. 10.1016/j.protcy.2016.05.007

[B5] Bertino L. Evensen G. Wackernagel H. (2003). Sequential Data Assimilation Techniques in Oceanography. Int. Stat. Rev. 71 (2), 223–241. 10.1111/j.1751-5823.2003.tb00194.x

[B6] Bjerklie D. M. Birkett C. M. Jones J. W. Carabajal C. Rover J. A. Fulton J. W. (2018). Satellite Remote Sensing Estimation of River Discharge: Application to the Yukon River Alaska. J. Hydrol. 561 (April), 1000–1018. 10.1016/j.jhydrol.2018.04.005

[B7] Blythe T. L. Schmidt J. C. (2018). Estimating the Natural Flow Regime of Rivers with Long-Standing Development: The Northern Branch of the Rio Grande. Water Resour. Res. 54 (2), 1212–1236. 10.1002/2017wr021919

[B8] Cao Y. Ye Y. Liang L. Zhao H. Jiang Y. Wang H. (2019). A Modified Particle Filter‐Based Data Assimilation Method for a High‐Precision 2‐D Hydrodynamic Model Considering Spatial‐temporal Variability of Roughness: Simulation of Dam‐Break Flood Inundation. Water Resour. Res. 55, 6049–6068. 10.1029/2018wr023568

[B9] Chai T. Draxler R. R. (2014). Root Mean Square Error (RMSE) or Mean Absolute Error (MAE)? - Arguments against Avoiding RMSE in the Literature. Geosci. Model. Dev. 7 (3), 1247–1250. 10.5194/gmd-7-1247-2014

[B10] Crisan D. Doucet A. (2002). A Survey of Convergence Results on Particle Filtering Methods for Practitioners. IEEE Trans. Signal. Process. 50 (3), 736–746. 10.1109/78.984773

[B11] Ding D. Wang Z. Alsaadi F. E. Shen B. (2015). Receding Horizon Filtering for a Class of Discrete Time-Varying Nonlinear Systems with Multiple Missing Measurements. Int. J. Gen. Syst. 44 (2), 198–211. 10.1080/03081079.2014.973732

[B12] Feng J. Wang Z. Zeng M. (2011). Recursive Robust Filtering with Finite-step Correlated Process Noises and Missing Measurements. Circuits Syst. Signal. Process. 30 (6), 1355–1368. 10.1007/s00034-011-9289-6

[B13] Gadsden S. A. Al-Shabi M. Arasaratnam I. Habibi S. R. (2014). Combined Cubature Kalman and Smooth Variable Structure Filtering: A Robust Nonlinear Estimation Strategy. Signal. Process. 96 (PART B), 290–299. 10.1016/j.sigpro.2013.08.015

[B14] Gadsden S. A. Habibi S. R. Kirubarajan T. (2012). The Smooth Particle Variable Structure Filter. Trans. Can. Soc. Mech. Eng. 36 (2), 177–193. 10.1139/tcsme-2012-0013

[B15] Habibi S. (2007). The Smooth Variable Structure Filter. Proc. IEEE 95 (5), 1026–1059. 10.1109/jproc.2007.893255

[B16] He X. Sithiravel R. Tharmarasa R. Balaji B. Kirubarajan T. (2014). A Spline Filter for Multidimensional Nonlinear State Estimation. Signal. Process. 102, 282–295. 10.1016/j.sigpro.2014.03.051

[B17] Hu J. Wang Z. Gao H. Stergioulas L. K. (2012). Extended Kalman Filtering with Stochastic Nonlinearities and Multiple Missing Measurements. Automatica 48 (9), 2007–2015. 10.1016/j.automatica.2012.03.027

[B18] Ismail Z. H. Jalaludin N. A. (2016). River Flow and Stage Estimation With Missing Observation Data Using Multi Imputation Particle Filter (MIPF) Method. J. Telecommun. Electron. Comput. Eng. (JTEC) 8 (11), 145–150.

[B19] Jain S. K. Mani P. Jain S. K. Prakash P. Singh V. P. Tullos D. (2018). A Brief Review of Flood Forecasting Techniques and Their Applications. Int. J. River Basin Manage. 16 (3), 329–344. 10.1080/15715124.2017.1411920

[B20] Kang H. (2013). The Prevention and Handling of the Missing Data. Korean J. Anesthesiol. 64 (5), 402–406. 10.4097/kjae.2013.64.5.402 23741561 PMC3668100

[B21] Kim S. Seo D.-J. Riazi H. Shin C. (2014). Improving Water Quality Forecasting via Data Assimilation – Application of Maximum Likelihood Ensemble Filter to HSPF. J. Hydrol. 519, 2797–2809. 10.1016/j.jhydrol.2014.09.051

[B22] Lee H.-C. Lin C.-Y. Lin C.-H. Hsu S.-W. King C.-T. (2011). A Low-Cost Method for Measuring Surface Currents and Modeling Drifting Objects. IEEE Trans. Instrum. Meas. 60 (3), 980–989. 10.1109/tim.2010.2062730

[B23] Li D. Z. Wang W. Ismail F. (2014). A Mutated Particle Filter Technique for System State Estimation and Battery Life Prediction. IEEE Trans. Instrum. Meas. 63 (8), 2034–2043. 10.1109/tim.2014.2303534

[B24] Liang K. Lingfu K. Peiliang W. U. (2012). “Adaptive Gaussian Particle Filter for Nonlinear State Estimation,” in 31st Chinese Control Conference, Hefei, China, July 25-27, 2012, 2146–2150.

[B25] Litrico X. Fromion V. (2009). “Modeling of Open Channel Flow,” in Modeling and Control of Hydrosystems. 1st ed. (London, UK: Springer-Verlag), 17–41.

[B26] Liu W.-Q. Wang X.-M. Deng Z.-L. (2017). Robust Centralized and Weighted Measurement Fusion Kalman Estimators for Uncertain Multisensor Systems with Linearly Correlated white Noises. Inf. Fusion 35, 11–25. 10.1016/j.inffus.2016.08.002

[B27] Liu Y. Gupta H. V. (2007). Uncertainty in Hydrologic Modeling: Toward an Integrated Data Assimilation Framework. Water Resour. Res. 43, 1–18. 10.1029/2006wr005756 20300476

[B28] Mao J. Ding D. Song Y. Liu Y. Alsaadi F. E. (2017). Event-based Recursive Filtering for Time-Delayed Stochastic Nonlinear Systems with Missing Measurements. Signal. Process. 134, 158–165. 10.1016/j.sigpro.2016.12.004

[B29] Mukherjee A. Sengupta A. (2010). Likelihood Function Modeling of Particle Filter in Presence of Non-stationary Non-gaussian Measurement Noise. Signal. Process. 90 (6), 1873–1885. 10.1016/j.sigpro.2009.12.005

[B30] Ogundijo O. E. Elmas A. Wang X. (2016). Reverse Engineering Gene Regulatory Networks from Measurement with Missing Values. J. Bioinform Sys Biol. 2017 (2017), 2. 10.1186/s13637-016-0055-8 PMC5225239 28127303

[B31] Pintelon R. Ugryumova D. Vandersteen G. Louarroudi E. Lataire J. (2017). Time-Variant Frequency Response Function Measurement in the Presence of Missing Data. IEEE Trans. Instrum. Meas. 66 (11), 3091–3099. 10.1109/tim.2017.2728218

[B32] Rafieeinasab A. Seo D.-J. Lee H. Kim S. (2014). Comparative Evaluation of Maximum Likelihood Ensemble Filter and Ensemble Kalman Filter for Real-Time Assimilation of Streamflow Data into Operational Hydrologic Models. J. Hydrol. 519, 2663–2675. 10.1016/j.jhydrol.2014.06.052

[B33] Rigatos G. G. (2012). A Derivative-free Kalman Filtering Approach to State Estimation-Based Control of Nonlinear Systems. IEEE Trans. Ind. Electron. 59 (10), 3987–3997. 10.1109/tie.2011.2159954

[B34] Sichangi A. W. Wang L. Hu Z. (2018). Estimation of River Discharge Solely from Remote-Sensing Derived Data: An Initial Study over the Yangtze River. Remote Sens. 10 (9). 10.3390/rs10091385

[B35] Smith P. J. Thornhill G. D. Dance S. L. Lawless A. S. Mason D. C. Nichols N. K. (2013). Data Assimilation for State and Parameter Estimation: Application to Morphodynamic Modelling. Q.J.R. Meteorol. Soc. 139 (671), 314–327. 10.1002/qj.1944

[B36] Solonen A. Hakkarainen J. Ilin A. Abbas M. Bibov A. (2014). Estimating Model Error Covariance Matrix Parameters in Extended Kalman Filtering. Nonlin. Process. Geophys. 21 (5), 919–927. 10.5194/npg-21-919-2014

[B37] Tarpanelli A. Santi E. Tourian M. J. Filippucci P. Amarnath G. Brocca L. (2019). Daily River Discharge Estimates by Merging Satellite Optical Sensors and Radar Altimetry through Artificial Neural Network. IEEE Trans. Geosci. Remote Sensing 57 (1), 329–341. 10.1109/tgrs.2018.2854625

[B38] Tinka A. Rafiee M. Bayen A. M. (2013). Floating Sensor Networks for River Studies. IEEE Syst. J. 7 (1), 36–49. 10.1109/jsyst.2012.2204914

[B39] Ugryumova D. Pintelon R. Vandersteen G. Member S. (2015). Frequency Response Function Estimation in the Presence of Missing Output Data. IEEE Trans. Instrum. Meas. 64 (2), 541–553. 10.1109/tim.2014.2342431

[B40] Wang S. Fang H. Tian X. (2017). Robust Estimator Design for Networked Uncertain Systems with Imperfect Measurements and Uncertain-Covariance Noises. Neurocomputing 230, 40–47. 10.1016/j.neucom.2016.11.035

[B41] Zhang X.-P. Khwaja A. S. Luo J.-A. Housfater A. S. Anpalagan A. (2015). Multiple Imputations Particle Filters: Convergence and Performance Analyses for Nonlinear State Estimation with Missing Data. IEEE J. Sel. Top. Signal. Process. 9 (8), 1536–1547. 10.1109/jstsp.2015.2465360

[B42] Zhang X. Khwaja A. S. Luo J. Housfater A. S. Anpalagan A. (2014). Convergence Analysis of Multiple Imputations Particle Filters for Dealing with Missing Data in Nonlinear Problems. IEEE J. Sel. Top. Signal. Process. 9 (8), 2567–2570. 10.1109/iscas.2014.6865697

